# The Arabidopsis *ELP3*/*ELO3* and *ELP4*/*ELO1* genes enhance disease resistance in *Fragaria vesca* L.

**DOI:** 10.1186/s12870-017-1173-5

**Published:** 2017-12-01

**Authors:** Katchen Julliany P. Silva, Asha M. Brunings, Juliana A. Pereira, Natalia A. Peres, Kevin M. Folta, Zhonglin Mou

**Affiliations:** 1 Department of Microbiology and Cell Science, University of Florida, Gainesville, FL 32611 USA; 2 Department of Horticultural Sciences, University of Florida, Gainesville, FL 32611 USA; 3 Department of Plant Pathology, Gulf Coast Research and Education Center, University of Florida, Wimauma, FL 33598 USA; 4 Department of Plant Pathology, University of Florida, Gainesville, FL 32611 USA

**Keywords:** *Fragaria vesca* L., Disease resistance, The Elongator complex, *AtELP3*, *AtELP4*, *FvELP4*, Transgenic plants

## Abstract

**Background:**

Plant immune response is associated with a large-scale transcriptional reprogramming, which is regulated by numerous transcription regulators such as the Elongator complex. Elongator is a multitasking protein complex involved in diverse cellular processes, including histone modification, DNA methylation, and tRNA modification. In recent years, Elongator is emerging as a key regulator of plant immune responses. However, characterization of Elongator’s function in plant immunity has been conducted only in the model plant *Arabidopsis thaliana*. It is thus unclear whether Elongator’s role in plant immunity is conserved in higher plants. The objective of this study is to characterize transgenic woodland strawberry (*Fragaria vesca* L.) overexpressing the Arabidopsis Elongator (AtELP) genes, *AtELP3* and *AtELP4*, and to determine whether *F. vesca* carries a functional Elongator complex.

**Methods:**

Transgenic *F. vesca* and Arabidopsis plants were produced via Agrobacterium-mediated genetic transformation and characterized by morphology, PCR, real-time quantitative PCR, and disease resistance test. The Student’s *t* test was used to analyze the data.

**Results:**

Overexpression of *AtELP3* and *AtELP4* in *F. vesca* impacts plant growth and development and confers enhanced resistance to anthracnose crown rot, powdery mildew, and angular leaf spot, which are caused by the hemibiotrophic fungal pathogen *Colletotrichum gloeosporioides*, the obligate biotrophic fungal pathogen *Podosphaera aphanis*, and the hemibiotrophic bacterial pathogen *Xanthomonas fragariae*, respectively. Moreover, the *F. vesca* genome encodes all six Elongator subunits by single-copy genes with the exception of FvELP4, which is encoded by two homologous genes, *FvELP4–1* and *FvELP4–2*. We show that *FvELP4–1* complemented the Arabidopsis *Atelp4*/*elo1–1* mutant, indicating that FvELP4 is biologically functional.

**Conclusions:**

This is the first report on overexpression of Elongator genes in plants. Our results indicate that the function of Elongator in plant immunity is most likely conserved in *F. vesca* and suggest that Elongator genes may hold potential for helping mitigate disease severity and reduce the use of fungicides in strawberry industry.

**Electronic supplementary material:**

The online version of this article (10.1186/s12870-017-1173-5) contains supplementary material, which is available to authorized users.

## Background

Both plants and animals are constantly exposed to potential microbial pathogens. However, unlike animals, plants lack adaptive immunity and rely solely on the innate immune system to defend themselves against microbial invasion. The plant immune system detects microbial invasion by recognizing the invasion patterns (IPs) generated during plant-microbe interaction [1]. IPs, including the well-described microbe/pathogen-associated molecular patterns, damage-associated molecular patterns, and effectors, are perceived by plant IP receptors (IPRs), such as receptor-like kinases, receptor-like proteins, and nucleotide-binding domain and leucine-rich repeat-containing proteins [2]. IP-IPR interaction locally triggers immunity against the invading pathogen and also distally induces systemic acquired resistance (SAR) in non-infected leaf tissues, which confers long-lasting protection against subsequent infection by a broad spectrum of pathogens [3].

The establishment of plant immunity is associated with profound transcriptional reprogramming in plant cells [4, 5]. The efficacy of the immunity is tightly correlated with the kinetics and magnitude of the transcriptional changes. A large body of genetic evidence has demonstrated that suppression and/or delay of pathogen-induced transcriptional changes by pathogen effectors or mutations in immune-related components compromise plant immunity [2, 5, 6]. It is thus vital for plant cells to rapidly yet precisely reprogram their transcriptome in response to pathogen invasion.

Numerous regulators have been shown to modulate plant immune-associated transcriptional reprogramming, among which is the multitasking protein complex named Elongator. Elongator was initially purified as an interactor of elongating RNA polymerase II in yeast, and subsequently was identified in animal and plant cells [7–9]. The Elongator complex is composed of two copies of each of its six subunits (ELP1 to ELP6). ELP1 and ELP2 are scaffolds for complex assembly, ELP3 is the catalytic subunit, and ELP4-ELP6 form an accessory complex*.* It has been reported that Elongator functions in diverse cellular processes, including histone modification, exocytosis, α-tubulin acetylation, transcriptional silencing, genome stability maintenance, DNA methylation and/or demethylation, tRNA modification, and microRNA biogenesis [10–16]*.* Interestingly, Elongator plays kingdom-specific roles in distinct organisms [17]*.* For instance, yeast Elongator mutants exhibit resistance to the zymocin γ-toxin and sensitivity to salt, caffeine, and temperature [7, 18, 19], whereas human Elongator deficiency leads to defective neuron development, evinced as familial dysautonomia disease [20, 21]*.*

The Arabidopsis Elongator (AtELP) complex has been well characterized and its six subunits, AtELP1/ELONGATA2 (ELO2), AtELP2, AtELP3/ELO3, AtELP4/ELO1, AtELP5, and AtELP6, have been defined [9, 22]. *Atelp*/*elo* mutants display pleiotropic phenotypes, including hypersensitivity to abscisic acid, resistance to oxidative stress, severely aberrant auxin phenotypes, altered cell cycle progression, abnormal root development, and disease susceptibility [6, 9, 23–29]. Recent studies have shown that AtELP2 and AtELP3 regulate the kinetics of pathogen-induced transcriptome reprogramming [6, 30]*.* In-depth investigation revealed that AtELP2 regulates pathogen-induced transcriptome changes likely through maintaining histone acetylation levels, modulating the genomic DNA landscape, and influencing pathogen-induced dynamic DNA methylation changes [31]*.*

Besides the Arabidopsis Elongator complex, the tomato *AtELP2* ortholog, *SlELP2L*, was recently characterized [32]. *SlELP2L*-RNAi transgenic tomato plants exhibit pleiotropic phenotypes, including delayed seedling development, reduced leaf growth, rapidly senescing leaves and sepals, and dark-green fruit, which are reminiscent of Arabidopsis *Atelp*/*elo* mutants. However, some of the *SlELP2L*-RNAi phenotypes are in sharp contrast to those of *Atelp*/*elo* mutant plants. For example, ethylene signaling is enhanced in *Atelp*/*elo* plants [9], but suppressed in *SlELP2L*-RNAi tomato plants [32]. Furthermore, *Atelp*/*elo* mutants produce high levels of auxin [9], whereas *SlELP2L*-RNAi tomato plants accumulate decreased levels of auxin [32]. These phenotypic differences suggest that the function of Elongator in different plant species may not be fully conserved.

In this study, we transformed *AtELP3* and *AtELP4* into the woodland strawberry *Fragaria vesca* L. and characterized the resulting transgenic plants. Our results show that overexpression of *AtELP3* and *AtELP4* in *F. vesca* influences plant growth and development and confers resistance to the obligate biotrophic fungal pathogen *Podosphaera aphanis*, the hemibiotrophic fungal pathogen *Colletotrichum gloeosporioides*, and the hemibiotrophic bacterial pathogen *Xanthomonas fragariae*. These results underscore the important emerging role of Elongator in plant immunity [33]. Our results also indicate that the *F. vesca* genome encodes all six Elongator subunits. We show that FvELP4 complements the morphological phenotypes of the Arabidopsis *Atelp4*/*elo1–1* mutant, indicating that it is biologically functional. Taken together, our results suggest that the function of the Elongator complex is likely conserved in strawberry.

## Methods

### Plasmid construction and plant transformation

The coding regions of *AtELP3* and *AtELP4* were amplified from cDNAs by PCR and cloned into the Gateway T-DNA vector pK7WG2D,1, which contains a neomycin phosphotransferase II (*nptII*) gene for plant selection and an enhanced green fluorescent protein (GFP) reporter for visual selection. The resulting plasmids were introduced into the *Agrobacterium* strain GV3101 by electrophoresis, which was then used for genetic transformation of the diploid woodland strawberry *F. vesca* L. Leaf explants of *F. vesca* accession ‘Hawaii-4’ were transformed using an optimized regeneration protocol [34, 35]. The accession ‘Hawaii-4’ is freely available without requiring a Material Transfer Agreement from the National Clonal Germplasm Repository accession # PI1551572. Transgenic calli and shoots were visually screened for GFP six weeks after co-culture, and well-developed and rooted shoots were transplanted into soil and placed in a growth chamber with a 12-h photoperiod. The plants were considered to be independent transgenic lines when regenerated from independent calli. ‘Hawaii-4’ seedlings, also derived through leaf regeneration, were used as a control in all experiments of this study. Plants were propagated by runners or by crown division, and watered and fertilized as needed. Pesticides were applied as necessary to control insects and mites. After two months of growth in soil, well-developed plants were used for experiments.

For complementation of the Arabidopsis *Atelp4*/*elo1–1* mutant, the coding region of *FvELP4–1* was amplified from *F. vesca* cDNA and cloned into the binary vector pBI1.4 T. The resulting plasmid was introduced into the *Agrobacterium* strain GV3101 by electrophoresis. Arabidopsis plant transformation was conducted following the floral dip method [36].

### SA measurement

SA content in *F. vesca* leaf tissues was measured by HPLC as described by Verberne et al. [37]. Briefly, 100 mg tissues were ground in liquid nitrogen and extracted with 1 mL of 90% methanol. After centrifugation at 14,000 g for 10 min, the supernatant was transferred into a microcentrifuge tube. The pellet was extracted with 0.5 mL of 100% methanol and the supernatant was transferred to the same tube and dried in a speed vacuum to final volume of ~50 μL. The residue was resuspended to 500 μL sodium acetate buffer (0.2 M, pH 5.5). After centrifugation at 14,000 g for 10 min, the supernatant was used for HPLC analysis. The sample was eluted with 0.2 M sodium acetate buffer pH 5.5 in 10% methanol at a flow-rate of 0.80 mL/min.

### Assessment of disease resistance

To evaluate the effect of *AtELP3* and *AtELP4* on disease resistance, we used the following pathogens: *C. gloeosporioides*, *P. aphanis*, and *X. fragariae*, which are causal agents of strawberry anthracnose crown rot, powdery mildew, and angular leaf spot, respectively. The pathogen isolates used in this study were obtained from plants grown in the greenhouse or strawberry fields located in West Central Florida. *C. gloeosporioides* and *X. fragariae* isolates were stored at −80°C in 20% glycerol, and *P. aphanis* was stored in the Herbarium and Culture Collection at the University of Florida Gulf Coast Research and Education Center (GCREC).

Three isolates of *C. gloeosporioides* (CG#13–01, GG#98–285 and CG#97-15A) used in this study were obtained from diseased strawberry crowns. Colonies were maintained on potato-dextrose-agar (PDA) for 6 to 8 days at 24 °C. Conidial suspension and plant inoculation were performed as described previously [35]. Briefly, a conidial suspension (1 × 10^6^ conidia mL^−1^) was prepared for each isolate and the three suspensions were then combined. Inoculations were performed by spraying 2 to 3 mL of conidial suspension onto the crown and canopy of five plants per treatment using an atomizer. Plants were placed into plastic boxes to maintain 90–100% relative humidity (RH) and 20.6 ± 0.5 °C. After 72 h, the boxes were removed and the plants were kept at the same temperature and 60 ± 5% RH.

The number of individual plants that collapsed at 20 days post-inoculation was used to determine disease incidence (DI). DI was also assessed every 2 days, allowing the calculation of the area under the disease progress curve (AUDPC) [38]: $\mathrm{AUDPC}=\sum \limits_{i=1}^{Ni=1}\frac{\left({y}_i+{y}_{i+1}\right)}{2}\left({t}_{i+1}-{t}_i\right)$, where N_i_ is number of assessments; (*y*_*i*_ + *y*_*i* + 1_) is the sum of initial and consecutive disease incidence; and (*t*_*i* + 1_ − *t*_*i*_) is the time interval between two consecutive assessments. The experiment was conducted with five plants per *F. vesca* line each time.

Powdery mildew (PM#15–31) was originally obtained from the cultivar ‘Strawberry Festival’ (*Fragaria x ananasa* Duch.) and identified as *P. aphanis* based on the conidiophores and/or chasmothecia detected on leaf surfaces. Non-transformed ‘Hawaii-4’ plants were initially used for the production of inoculum. Inoculation was performed by physically rubbing diseased leaves on both abaxial and adaxial surfaces of all leaves on each non-transformed and transgenic strawberry plant. Inoculated plants were kept in growth chamber at 80% RH and 22 °C for 8 days. After incubation, the plants were assigned disease reaction (DR) scores according to the method described by Gollner et al. [39], where a DR score of 2 describes fully susceptible plants, indicating that extensive pathogen growth was observed; a DR score of 0 refers to fully resistant plants, on which no fungal structure and disease symptoms could be observed; and a DR score of 1 denotes plants with intermediate susceptibility, which show fungal structures on less than 10% of the leaf surface. Spore counting was performed following the method described by Silva et al. [35]. Briefly, 8 days after inoculation, three leaflets were randomly taken from the plant canopy and three samples of approximately 500 mg of leaf tissues were harvested per genotype and transferred to microcentrifuge tubes containing 1 mL of distilled water. Spores were liberated by vortexing for 30 s at maximum speed. After filtering through miracloth, spores were counted using a haemocytometer. Spore counts were normalized against the initial sample weight. The experiment was done with five plants per line each time.

The bacterial pathogen *X. fragariae* (XF#11–15) was initially isolated from leaves of ‘Strawberry Festival’ and kept on solid sucrose-peptone agar (SPA) (5% peptone, 0.5% K_2_HPO_4_, 0.25% MgSO_4_.7H_2_O, 10% sucrose and 18% agar) at ±29 °C for 4 days. Suspension preparation and inoculation were performed according to Silva et al. [35], which was adapted from Maas et al. [40]. Briefly, bacteria was washed from the SPA plates with sterile distilled water and the suspensions were diluted to a final concentration of approximately 10^9^ colony forming units (CFU) mL^−1^. Inoculation was performed by firmly placing the aperture of a 3-mL needleless syringe against the abaxial surface of a leaflet. The syringe plunger was carefully depressed until a water-soaked area became visible. Each leaflet was inoculated at four sites away from the midrib, totaling 12 inoculations per leaf. Inoculated plants were placed into plastic containers and kept in a growth chamber (22 °C, 16 h light photoperiod, 70 ± 5% RH) for four weeks.

The inoculation sites were evaluated and rated at the end of the fourth week using the following scale: 0 = no reaction, transient water-soaking from inoculation no longer evident; 1 = transient water-soaking evident in the inoculation site; 2 = slight chlorosis or necrosis in the center of the inoculation site; 3 = water-soaking expanding beyond inoculation site and often bacterial exudate evident; 4 = necrosis spreading beyond the inoculation site and/or secondary infections evident; and 5 = total necrosis of the inoculation area and leaflet changing color from chlorotic to reddish-brown. Plants were considered susceptible on a whole-plant basis if bacterial exudate was produced at any inoculation site, if inoculation sites remained translucent, or if secondary infection sites were apparent on the inoculated leaves. Plants were considered resistant if inoculation sites lost translucency and/or developed necrotic centers that did not progress beyond the inoculation sites.

At the end of the experiment, the inoculation sites were cut from the leaflets and surface sterilized using 70% ethanol and 10% bleach for 1 min. The leaf discs were then washed three times with sterile distilled water, surface dried with filter paper, and transferred to microcentrifuge tubes containing 1 mL of sterile water. The leaf tissues were ground with a polypropylene pellet pestle, vortexed, and serially diluted. Finally, 100 μL of the dilutions were transferred onto solid SPA in culture plates, which were then incubated at room temperature for *X. fragariae* colony development [41].

### PCR and real-time qPCR analysis

*F. vesca* genomic DNA was extracted from leaf tissues using a CTAB method optimized for strawberry [42, 43]. The presence of the transgene in the transgenic plants was verified by PCR using gene-specific primers under the following conditions: 94 °C for 5 min, 35 cycles (94 °C for 3 min, 65 °C for 1 min, 72 °C for 2 min), and finally at 72 °C for 10 min. Total RNA was extracted using an RNeasy® Plant Mini-Kit (Qiagen) following the manufacturer’s instructions. One μg RNA was reverse transcribed using the Improm-II Reverse Transcriptase (Promega Inc., Madison, WI). Reverse transcription and RT-qPCR was performed using the StepOne Plus system (Applied Biosystems, USA) based on SYBR Green chemistry. Expression of *AtELP3*, *AtELP4*, *FvPR1*, and *FvNPR5* was normalized against the strawberry elongation factor-1-alpha (*EF1α*) gene, and calculated using the formula 2^[Ct(*EF1α*)-Ct(*GENE)*]^ [44, 45]. We chose *EF1α* as the internal control for normalization, as we have previously shown that *EF1α* is one of the most stably expressed transcripts in *F. vesca* [46]. Primers used in this study are listed in Additional file 1: Table S1.

### Statistical analysis

Statistical analysis was conducted using the data analysis tools (Student’s *t* test: Two Samples Assuming Unequal Variances) in Microsoft Excel of Microsoft Office 2004 for Macintosh. All experiments were repeated at least three times with similar trends.

## Results

### Overexpression of *AtELP3* and *AtELP4* in *F. vesca* impacts plant growth and development

**Fig. 1:**
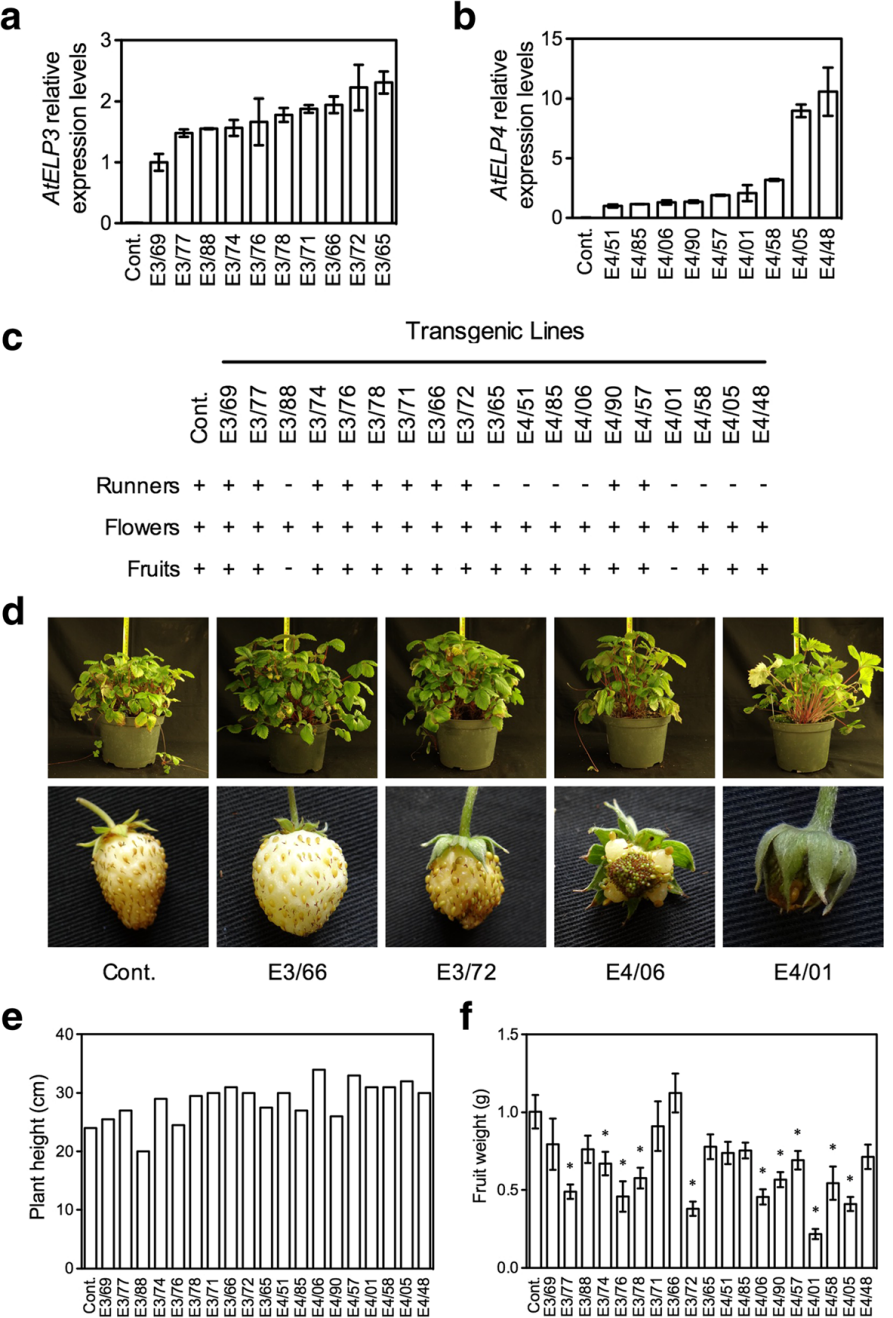
Molecular and morphological characterization of transgenic *F. vesca* plants expressing *AtELP3* and *AtELP4*. **a** and **b** Expression levels of *AtELP3* (**a**) and *AtELP4* (**b**) in independent *AtELP3* (E3) and *AtELP4* (E4) transgenic *F. vesca* lines. No *AtELP3* and *AtEL4* expression was detected in the non-transformed control (Cont.). The order of the transgenic lines is presented in order of increasing expression levels of the transgene. Expression of the transgene was normalized against the constitutively expressed *EF1α* gene. The resulting average values of E3/69 and E4/51 were arbitrarily set as 1 in (**a**) and (**b**), respectively, and other lines were compared with E3/69 or E4/51 to show the relative expression levels of the transgenes. Data represent the average of three biological replicates with standard deviation (SD). The experiments were repeated with similar trends. **c** Presence (+) or absence (−) of runners, flowers, and fruit on the *AtELP3* (E3) and *AtELP4* (E4) transgenic plants. The order of the transgenic lines is the same as in Fig. 1a and b. **d** Plant (top) and fruit (bottom) morphology of two *AtELP3* (E3/66 and E3/72) and two *AtELP4* (E4/06 and E4/01) transgenic lines as well as the non-transformed control (Cont.). **e** and **f** Plant height (**e**) and fruit weight (**f**) of one-year-old *AtELP3* (E3) and *AtELP4* (E4) transgenic plants. The fruit weight data in (**f**) represent the average of 20 strawberries with SD. An asterisk indicates significant difference between the transgenic line and the non-transformed control (Cont.) (Student’s *t*-test, *p* < 0.05)

To test if *AtELP3* and *AtELP4* function in strawberry, both genes were introduced into the diploid woodland strawberry *F. vesca* via Agrobacterium-mediated genetic transformation. Ten and nine transgenic *F. vesca* plants carrying *AtELP3* and *AtELP4*, respectively, were selected for further characterization. Real-time quantitative PCR (RT-qPCR) analysis revealed that *AtELP3* and *AtELP4* were expressed at different levels in the transgenic plants (Fig. 1a and b). All transgenic plants formed flowers, and one *AtELP3*-expressing plant (E3 plant, E3/88) and two *AtELP4*-expressing plants (E4 plants, E4/06 and E4/01) had aborted or irregular fruit (Fig. 1c and d). While the majority of E4 plants (except E4/90 and E4/57) did not produce runners, only two E3 plants (E3/88 and E3/65) did not do so (Fig. 1c). In general, E3 plants had a regular canopy, and E4 plants showed an elongated and less dense canopy compared with that of the control (Fig. 1d). All transgenic plants except E3/69, E3/88, and E3/76 were taller than the control plant (Fig. 1e), and most of the transgenic plants produced fruit significantly smaller than that of the control (Fig. 1f). These results indicate that overexpression of *AtELP3* or *AtELP4* in *F. vesca* affects plant growth and development.

### *F. vesca* Plants overexpressing *AtELP3* and *AtELP4* constitutively express defense genes

**Fig. 2:**
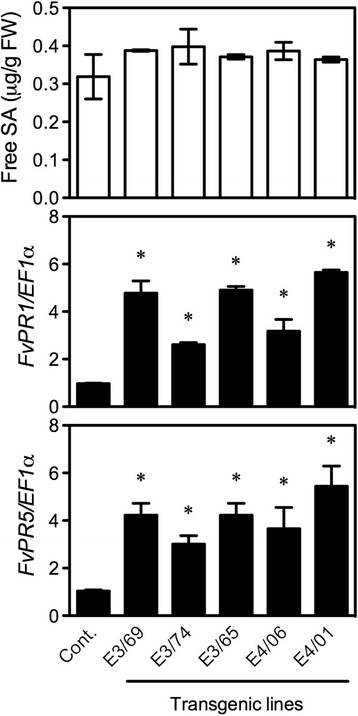
SA accumulation and *PR* gene expression in the *AtELP3* and *AtELP4* transgenic *F. vesca* plants. Free SA levels (top) as well as *FvPR1* (middle) and *FvPR2* (bottom) expression levels in three *AtELP3* (E3) and two *AtELP4* (E4) transgenic lines and the non-transformed control (Cont.). SA levels were measured by HPLC and *PR* gene expression was analyzed by RT-qPCR. Data represent the average of three biological replicates with SD. Expression of *PR* genes was normalized against the constitutively expressed *EF1α* gene. An asterisk indicates significant difference between the transgenic line and the non-transformed control (Student’s *t*-test, *p* < 0.05). The experiments were repeated three times with similar trends

Since Elongator plays an important role in Arabidopsis immunity [33], overexpression of *AtELP3* and *AtELP4* in *F. vesca* may influence its defense responses. To test this hypothesis, we chose three E3 lines (E3/69, E3/74, and E3/65) and two E4 lines (E4/06 and E4/01) for defense response analysis based on their ability to be propagated vegetatively. We first measured basal free SA levels in the transgenic lines. As shown Fig. 2 (top), free SA levels in the five transgenic lines were comparable to those in the control. We then analyzed the expression of *FvPR1* and *FvPR5*, the *F. vesca* orthologs of *AtPR1* and *AtPR5*, which are regulated by Elongator in Arabidopsis [6]. As shown in Fig. 2 (middle and bottom), transcription of both *FvPR1* and *FvPR5* was significantly enhanced in the E3 and E4 lines compared with that in the control. This result indicates that overexpression of *AtELP3* and *AtELP4* in *F. vesca* results in constitutive defense gene expression.

### *F. vesca* Plants overexpressing *AtELP3* and *AtELP4* show improved resistance to anthracnose crown rot

**Fig. 3:**
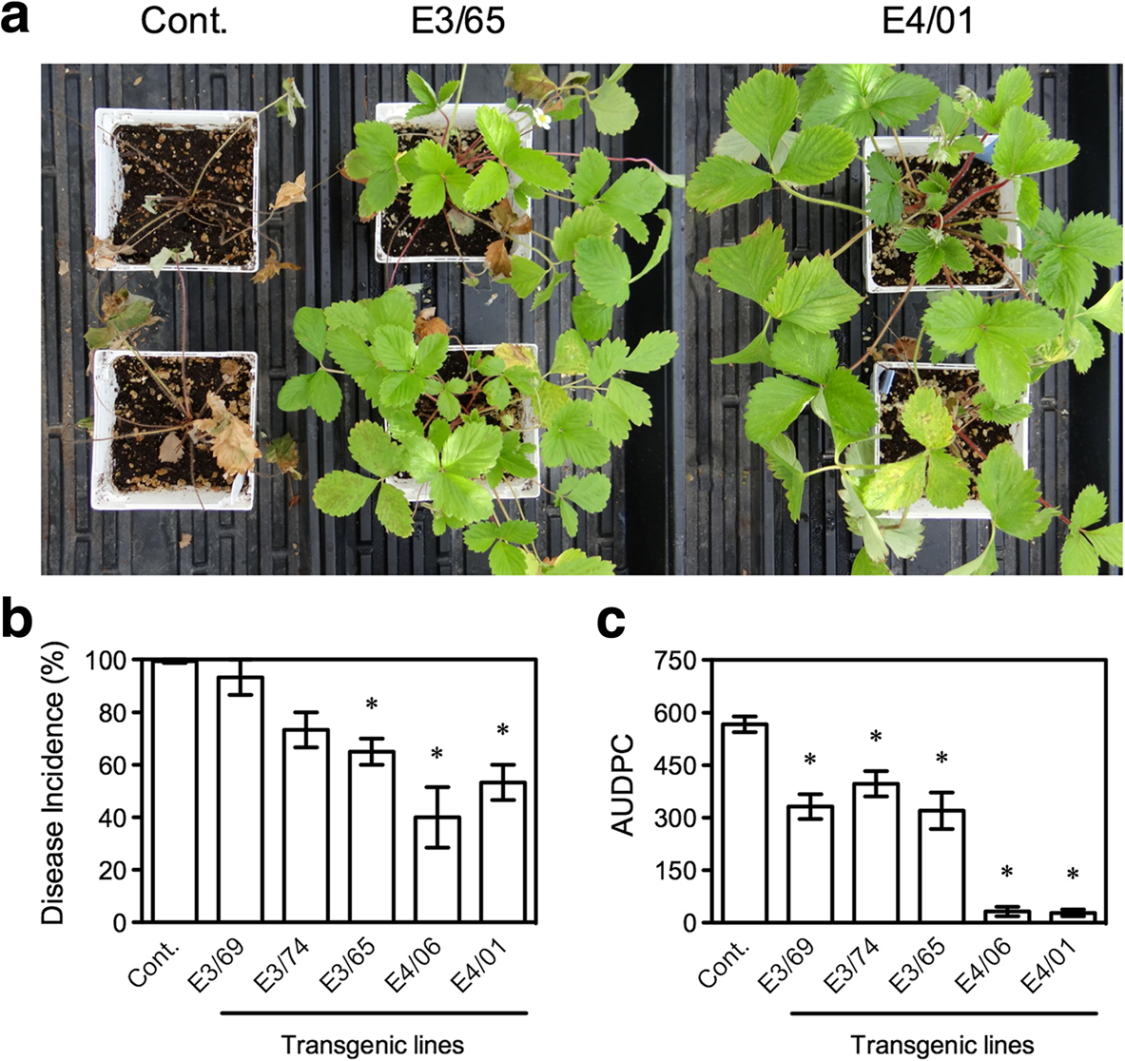
Resistance of the *AtELP3* and *AtELP4* transgenic *F. vesca* plants to anthracnose crown rot. **a** Disease symptoms caused by *C. gloeosporioides* on the transgenic lines E3/65 and E4/01 as well as the non-transformed control (Cont.). Photos were taken 20 days post-inoculation. **b** and **c** Disease incidence (DI, B) and the area under the disease progress curve (AUDPC, C) for the three *AtELP3* (E3) and two *AtELP4* (E4) transgenic lines and the non-transformed control (Cont.) infected with *C. gloeosporioides*. Data represent the average of collapsed plants in three independent experiments, each containing five plants per genotype, with SD. DI for AUDPC calculation was assessed every two days for 20 days. An asterisk indicates significant difference between the transgenic line and the non-transformed control (Student’s *t*-test, *p* < 0.05). The experiments were repeated four times with similar trends

Since the E3 and E4 plants constitutively express *FvPR1* and *FvPR5*, they may possess heightened resistance to pathogen infections. To test this possibility, we evaluated resistance of the transgenic plants to anthracnose crown rot caused by *C. gloeosporioides*. Crown rot symptoms, characterized by initial signs of water stress and subsequent plant collapse, were observed five days post-infection (dpi) on the control plants, which were then aggressively invaded by the pathogen and collapsed before 20 dpi, whereas the transgenic plants displayed reduced disease incidence (Fig. 3a). Disease incidence (DI), calculated as the percentage of diseased plants, was also significantly lower in E3/65, E4/06, and E4/01 than that in the control plants (Fig. 3b). While the DI was >99% in the control, it was 40% in the E4/06 plants. The resistance provided by *AtELP3* and *AtELP4* was further reflected by the area under the disease progress curve (AUDPC) (Fig. 3c). The AUDPCs of all the tested transgenic lines were significantly lower than those of the control plants. The two E4 lines, E4/06 and E4/01, exhibited extremely low AUDPCs (32 and 28, respectively), which were about 5.64% and 4.94%, respectively, of the control AUDPC (567) (Fig. 3c).

### *F. vesca* Plants overexpressing *AtELP3* and *AtELP4* exhibit enhanced resistance to powdery mildew

**Fig. 4:**
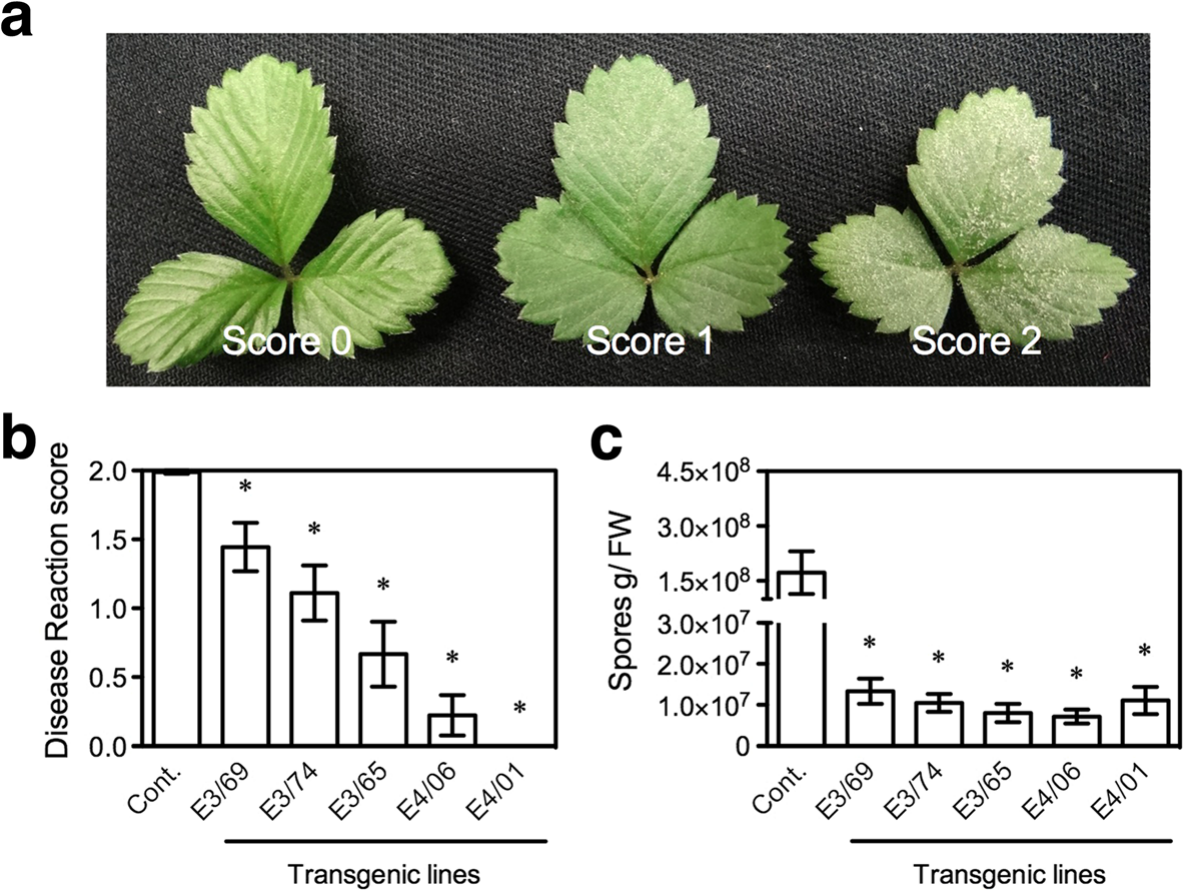
Resistance of the *AtELP3* and *AtELP4* transgenic *F. vesca* plants to powdery mildew. **a** Phenotypes of leaves to which different disease reaction scores were assigned after inoculation with *P. aphanis.* Score 0 = resistant, Score 1 = intermediate susceptibility, and Score 2 = susceptible. **b** Disease reaction scores for the three *AtELP3* (E3) and two *AtELP4* (E4) transgenic lines and the non-transformed control (Cont.). Data represent the average of nine biological replicates with SD. An asterisk indicates significant difference between the transgenic line and the non-transformed control (Student’s *t*-test, *p* < 0.05). **c** Growth of *P. aphanis* on the three *AtELP3* (E3) and two *AtELP4* (E4) transgenic lines and the non-transformed control (Cont.). Spores were counted 10 days after inoculation. FW: fresh weight. Data represent the average of 15 biological replicates with SD. An asterisk indicates significant difference between the transgenic line and the non-transformed control (Student’s *t*-test, *p* < 0.05). The experiments in (**b**) and (**c**) were repeated four times with similar trends

We also tested resistance of the transgenic *F. vesca* plants to powdery mildew, which is caused by the obligate biotrophic fungal pathogen *P. aphanis*. Eight days after *P. aphanis* inoculation, dense mycelial growth and numerous chains of conidia (spores) covered the entire surfaces of the leaves on the control plants with a disease reaction (DR) score of 2, indicating susceptibility to powdery mildew (Fig. 4a and b). In contrast, the transgenic line E4/01 was resistant (DR score = 0), and the transgenic lines E3/69, E3/74, E3/65, and E4/06 exhibited intermediate levels of resistance (DR scores between 0 and 2) (Fig. 4b), and allowed some mycelial growth. Furthermore, all tested transgenic lines had significantly fewer spores than the control plants (Fig. 4c). The number of spores on the transgenic plants was less than 8% of that on the control, indicating a strong resistance provided by *AtELP3* and *AtELP4*.

### *F. vesca* Plants overexpressing *AtELP3* and *AtELP4* display increased resistance to angular leaf spot

**Fig. 5:**
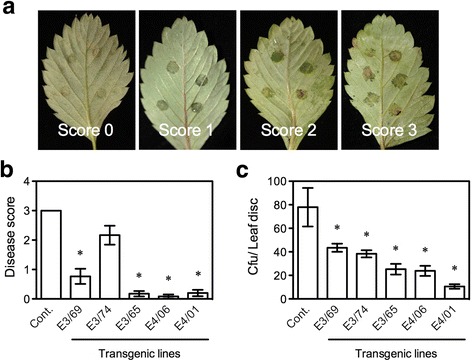
Resistance of the *AtELP3* and *AtELP4* transgenic *F. vesca* plants to angular leaf spot. **a** Responses at the inoculation sites to which different disease scores were assigned after inoculation with *X. fragariae*. Score 0: transient water-soaking from inoculation no longer evident; Score 1: transient water-soaking evident; Score 2: slight chlorosis or necrosis in the center of the inoculation site; and Score 3: water-soaking expanding beyond inoculation site with bacterial exudate often evident. **b** Disease scores of angular leaf spot on the three *AtELP3* (E3) and two *AtELP4* (E4) transgenic lines and the non-transformed control (Cont.). Data represent the average of 15 biological replicates with SD. An asterisk indicates significant difference between the transgenic line and the non-transformed control (Student’s *t*-test, *p* < 0.05). **c** Colony forming unit (CFU) of *X. fragariae* in the three *AtELP3* (E3) and two *AtELP4* (E4) transgenic lines and the non-transformed control (Cont.). Data represent the average of 15 biological replicates with SD. An asterisk indicates significant difference between the transgenic line and the non-transformed control (Student’s *t*-test, *p* < 0.05). The Experiments in (**b**) and (**c**) were repeated three times with similar trends

We further examined resistance of the transgenic lines to angular leaf spot, a disease caused by the bacterial pathogen *X. fragariae*. After syringe infiltration with *X. fragariae* suspensions, *F. vesca* leaves produced water-soaked lesions at the inoculation sites. Different disease scores were assigned to the distinct responses at the inoculation sites (Fig. 5a). At 7 dpi, the water-soaked lesions on control plants expanded beyond inoculation sites with visible necrosis and production of bacterial exudate (disease score = 3) (Fig. 5a and b). The transgenic line E3/74 developed slight necrosis in the center of the inoculation site (disease score > 2) (Fig. 5b). The inoculation sites on the transgenic lines E3/65, E4/06, and E4/01 lost translucency and did not develop any other symptom (disease scores close to 0) (Fig. 5b), and these lines were considered resistant to *X. fragariae*. An average disease score close to 1 was assigned to line E3/69, as the water-soaking phenotype of the inoculation sites was still evident on this line. To confirm the observed resistance in the transgenic plants, bacterial populations in the inoculated areas were determined. As shown in Fig. 5c, the bacterial populations were significantly lower in all the tested transgenic lines including E3/74, which exhibited symptoms, than in the control plants. The transgenic line E4/01 supported the lowest bacterial growth, with a bacterial population of only about 13.5% of that in the control. These results demonstrate that overexpression of *AtELP3* and *AtELP4* in *F. vesca* enhances resistance to the bacterial pathogen *X. fragariae.*

### The *F. vesca ELP4* gene encodes a functional protein

**Fig. 6:**
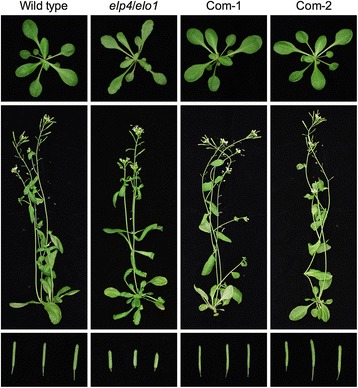
Complementation of the Arabidopsis *Atelp4* mutant by *FvELP4–1*. Morphological phenotypes of Arabidopsis wild type, *Atelp4*/*elo1–1*, and two independent complementation lines (*35S::FvELP4 Atelp4*), Com-1 and Com-2. Top: three-week-old plants; middle: six-week-old plants; bottom: siliques

Since the Elongator complex is highly conserved in eukaryotes [22], the *F. vesca* genome should contain genes encoding the six Elongator subunits. BLAST searches were conducted on the *X. fragariae* genome at Strawberry GARDEN (http://strawberry-garden.kazusa.or.jp/blast.html) using AtELP protein sequences as the queries. All genes encoding *F. vesca* Elongator (FvELP) subunits except *FvELP3* were annotated correctly, whereas *FvELP3* appears to be misannotated. After comparing with the *AtELP3* coding DNA sequence (CDS), we identified the likely correct CDS for *FvELP3* (Additional file 1: Figure S1). Additionally, there are two genetic loci (gene09242 and gene20701) encoding FvELP4, and FvELP4–1 (encoded by gene09242) is more similar to AtELP4 than FvELP4–2 (encoded by gene20701) (Additional file 1: Figure S2). Amino acid sequence alignments revealed that the FvELP proteins share high similarity (> 64%) with AtELPs (Additional file 1: Figure S2). To test whether FvELP proteins are functional, we introduced a *35S::FvELP4–1* transgene into the Arabidopsis *Atelp4*/*elo1–1* mutant via Agrobacterium-mediated genetic transformation. The *Atelp4*/*elo1–1* mutant morphological phenotypes, namely, narrow and elongated lamina, long petiole, and shortened siliques [23], were fully restored to the wild type in the transgenic plants (Fig. 6), indicating that the *FvELP4* gene is functional. Taken together, these results suggest that *F. vesca* most likely carries a functional Elongator complex.

## Discussion

By generating and characterizing transgenic *F. vesca* plants overexpressing the Arabidopsis *AtELP3* and *AtELP4* genes, here we show that: (1) overexpression of *AtELP3* and *AtELP4* in *F. vesca* affects plant growth and development; (2) overexpression of *AtELP3* and *AtELP4* in *F. vesca* increases resistance to two fungal and one bacterial pathogen; (3) the *F. vesca* genome encodes all six Elongator subunits; and (4) FvELP4 is biologically functional.

Elongator is a multitasking protein complex and mutations in Elongator lead to pleiotropic phenotypes including defects in plant growth, development, and immune responses [6, 23, 26, 27, 47]. The developmental and defense phenotypes of the transgenic *F. vesca* plants overexpressing *AtELP3* and *AtELP4* mirror the Elongator loss-of-function phenotypes. However, since constitutive defense responses often cause developmental alterations [48], it is not clear if the morphological changes of the *AtELP3* and *AtELP4* transgenic *F. vesca* plants are the result of heightened defense gene expression and disease resistance. Nevertheless, the *AtELP3* and *AtELP4* overexpression data generated in this study corroborate the emerging important role of Elongator in plant immunity [33].

It is intriguing that overexpression of a single Elongator subunit can enhance plant immune responses. The Elongator complex has been shown to function as a whole and loss of any subunit compromises Elongator integrity and renders the complex inactive [47, 49]. On the other hand, it has been reported that increasing *ELP3* expression in yeast suppresses the *anaphase-promoting complex5* mutant defects [50]. Moreover, overexpression of *ELP3* in human 293 T cells suppresses cell growth and enhances activated transcription [51]. Interestingly, although overexpression of *ELP4* alone in 293 T cells does not affect cell growth and transcription, overexpression of *ELP4* and *ELP3* together synergistically activates transcription [51]. In line with these reports, we found that overexpression of *AtELP3* and *AtELP4* in *F. vesca* elevates basal defense gene transcription and enhances disease resistance (Figs. 2, 3, 4, and 5). These results together suggest two independent but not mutually exclusive possibilities: (1) ELP3 and/or ELP4 may have some functions independent of the Elongator complex, and (2) ELP3 and/or ELP4 may somehow increase the Elongator complex activity.

However, it is not clear if overexpressing the other Elongator subunits including ELP1, ELP2, ELP5, and ELP6 will generate similar biological consequences. We chose to overexpress *AtELP3* and *AtELP4* because ELP3 is the catalytic subunit of the Elongator complex and *Atelp4* is the most disease-susceptible Arabidopsis Elongator mutant [23, 29, 52]. At this point, we cannot exclude the possibility that the four other Elongator subunits may also enhance disease resistance if overexpressed in plants. In this regard, generation and characterization of transgenic plants overexpressing *AtELP1*, *AtELP2*, *AtELP4*, and *AtELP6* will provide valuable information.

Overexpression of *AtELP3* and *AtELP4* may enhance defense gene transcription by optimizing chromatin structure [22, 33]. It has been shown that AtELP2 is associated with defense gene chromatin and that mutations in Elongator reduce histone acetylation levels and alter DNA methylation patterns in multiple defense genes [28, 29, 31]. It is conceivable to speculate that elevation of Elongator activity by overexpressing *AtELP3* and *AtELP4* would increase histone acetylation levels and change DNA methylation patterns, leading to heightened transcriptional responsiveness of defense genes. This speculation clearly deserves further investigation.

*F. vesca* likely contains a functional Elongator complex. Elongator is highly conserved both structurally and functionally in eukaryotes [22]. Indeed, the *F. vesca* genome has genes encoding each of the six Elongator subunits (Additional file 1: Figure S2). Whereas *FvELP1*, *FvELP2*, *FvELP3*, *FvELP5*, and *FvELP6* are single-copy genes, there are two copies of *FvELP4* (*FvELP4–1* and *FvELP4–2*). At the amino acid (AA) level, FvELP4–2 is 21 AAs longer than FvELP4–1 at the C-terminus and other AAs in the two proteins are 96% identical. The biological significance of this gene duplication is unknown. We show that *FvELP4–1* complemented the Arabidopsis *Atelp4*/*elo1–1* mutant (Fig. 6), indicating that FvELP4–1 is biologically functional.

Compared with the Arabidopsis *NPR1* (*AtNPR1*) gene, which encodes a key regulator of SAR [53], *AtELP3* and *AtELP4* appear to have less detrimental effects on *F. vesca* plant growth and development. For instance, when grown side by side, 90% and 89% of the *AtELP3* and *AtELP4* transgenic plants, respectively, produced fruit (Fig. 1c), whereas only 33% of the *AtNPR1* transgenic plants did so [35]. *AtELP3* and *AtELP4* also had milder effects on canopy size and density than *AtNPR1* (Fig. 1d) [35]. On the other hand, the disease resistance provided by *AtELP3* and *AtELP4* was comparable to, or even better than (in the case of anthracnose crown rot caused by *C. gloeosporioides*), that conferred by *AtNPR1* (Figs. 3, 4, and 5) [35]. As *AtNPR1* and its orthologs have been tested in many crop plants [35, 54–63], it would be interesting to investigate the performance of *AtELP3* and *AtELP4* as well as their orthologs in other crop species.

*C. gloeosporioides*, *P. aphanis*, and *X. fragariae* cause crown rot, powdery mildew, and angular leaf spot, respectively, in strawberry. *C. gloeosporioides* is present worldwide on multiple hosts and under favorable conditions causes massive plant death in nurseries and yield loss in strawberry production fields [64]. Powdery mildew is also widespread, predominantly causing foliar damage and also infecting fruit [65]. Angular leaf spot is potentially devastating in the strawberry industry and often found in commercial fruit production fields [66]. There is no cultivar that is entirely resistant to these pathogens. The transgenic *F. vesca* plants overexpressing *AtELP3* and *AtELP4* displayed increased resistance to the three pathogens (Figs. 3, 4, and 5). Several transgenic lines, including E3/65, E4/01, and E4/06, were highly resistant to *P. aphanis* and *X. fragariae* infection (Figs. 4 and 5). E4/01 and E4/06 were also highly resistant to *C. gloeosporioides* infection (Fig. 3). Although disease symptoms ultimately appeared on the transgenic plants, they were significantly delayed even under conditions that would favor disease occurrence. Because strawberries are grown as an annual crop, disease symptoms may not develop, or may be delayed until after major harvest periods on the transgenic plants.

## Conclusions

This is the first study on transgenic overexpression of Elongator genes in plants. Our results are in line with the emerging importance of the Elongator complex in plant immunity and indicate that the function of Elongator in plant immunity is most likely conserved in *F. vesca*. These results also suggest that *AtELP3* and *AtELP4* as well as their functional orthologs may hold the potential to mitigate disease symptoms and reduce the use of fungicides in strawberry production.

## Additional file

Additional file 1: Figure S1. Annotated and correct *FvELP3* CDS. **Figure S2.** Amino acid alignments between AtELPs and FvELPs. **Table S1.** Primers used in this study. (DOCX 260 kb)

## Abbreviations

AtELP: *Arabidopsis thaliana* Elongator complex protein
AtNPR1: *Arabidopsis thaliana* nonexpressor of pathogenesis-related genes1
AUDPC: area under the disease progress curve
CFU: colony forming unit
DI: disease incidence
DR: disease reaction
EF1α: elongation factor-1-alpha
ELO1: ELONGATA1
FvELP: *F. vesca* Elongator protein
FvPR: *F. vesca* pathogenesis-related protein
IPRs: IP receptors
IPs: invasion patterns
RT-qPCR: real-time quantitative PCR
SA: salicylic acid
SAR: systemic acquired resistance
SlELP2L: *Solanum lycopersicum* Elongator complex protein 2-like
SPA: sucrose peptone agar

## References

[1] Cook DE, Mesarich CH, Thomma BP (2015). Understanding plant immunity as a surveillance system to detect invasion. Annu Rev Phytopathol.

[2] Jones JD, Dangl JL (2006). The plant immune system. Nature.

[3] Durrant WE, Dong X (2004). Systemic acquired resistance. Annu Rev Phytopathol.

[4] Maleck K, Levine A, Eulgem T, Morgan A, Schmid J, Lawton KA, Dangl JL, Dietrich RA (2000). The transcriptome of *Arabidopsis thaliana* during systemic acquired resistance. Nature Genet.

[5] Tao Y, Xie Z, Chen W, Glazebrook J, Chang HS, Han B, Zhu T, Zou G, Katagiri F (2003). Quantitative nature of Arabidopsis responses during compatible and incompatible interactions with the bacterial pathogen *Pseudomonas syringae*. Plant Cell.

[6] Defraia CT, Zhang X, Mou Z (2010). Elongator subunit 2 is an accelerator of immune responses in *Arabidopsis thaliana*. Plant J.

[7] Otero G, Fellows J, Li Y, de Bizemont T, Dirac AM, Gustafsson CM, Erdjument-Bromage H, Tempst P, Svejstrup JQ (1999). Elongator, a multisubunit component of a novel RNA polymerase II holoenzyme for transcriptional elongation. Mol Cell.

[8] Hawkes NA, Otero G, Winkler GS, Marshall N, Dahmus ME, Krappmann D, Scheidereit C, Thomas CL, Schiavo G, Erdjument-Bromage H, Tempst P, Svejstrup JQ (2002). Purification and characterization of the human elongator complex. J Biol Chem.

[9] Nelissen H, De Groeve S, Fleury D, Neyt P, Bruno L, Bitonti MB, Vandenbussche F, Van der Straeten D, Yamaguchi T, Tsukaya H, Witters E, De Jaeger G, Houben A, Van Lijsebettens M (2010). Plant Elongator regulates auxin-related genes during RNA polymerase II transcription elongation. Proc Natl Acad Sci U S A.

[10] Winkler GS, Kristjuhan A, Erdjument-Bromage H, Tempst P, Svejstrup JQ (2002). Elongator is a histone H3 and H4 acetyltransferase important for normal histone acetylation levels in vivo. Proc Natl Acad Sci U S A.

[11] Huang B, Johansson MJ, Bystrom AS (2005). An early step in wobble uridine tRNA modification requires the Elongator complex. RNA.

[12] Rahl PB, Chen CZ, Collins RN (2005). Elp1p, the yeast homolog of the FD disease syndrome protein, negatively regulates exocytosis independently of transcriptional elongation. Mol Cell.

[13] Creppe C, Malinouskaya L, Volvert ML, Gillard M, Close P, Malaise O, Laguesse S, Cornez I, Rahmouni S, Ormenese S, Belachew S, Malgrange B, Chapelle JP, Siebenlist U, Moonen G, Chariot A, Nguyen L (2009). Elongator controls the migration and differentiation of cortical neurons through acetylation of alpha-tubulin. Cell.

[14] Li Q, Fazly AM, Zhou H, Huang S, Zhang Z, Stillman B (2009). The Elongator complex interacts with PCNA and modulates transcriptional silencing and sensitivity to DNA damage agents. PLoS Genet.

[15] Okada Y, Yamagata K, Hong K, Wakayama T, Zhang Y (2010). A role for the elongator complex in zygotic paternal genome demethylation. Nature.

[16] Fang X, Cui Y, Li Y, Qi Y (2015). Transcription and processing of primary microRNAs are coupled by Elongator complex in *Arabidopsis*. Nat Plants.

[17] Defraia CT, Mou Z (2011). The role of the Elongator complex in plants. Plant Signal Behav.

[18] Jablonowski D, Frohloff F, Fichtner L, Stark MJ, Schaffrath R (2001). *Kluyveromyces lactis* zymocin mode of action is linked to RNA polymerase II function via Elongator. Mol Microbiol.

[19] Krogan NJ, Greenblatt JF (2001). Characterization of a six-subunit holo-elongator complex required for the regulated expression of a group of genes in Saccharomyces Cerevisiae. Mol Cell Biol.

[20] Anderson SL, Coli R, Daly IW, Kichula EA, Rork MJ, Volpi SA, Ekstein J, Rubin BY (2001). Familial dysautonomia is caused by mutations of the IKAP gene. Am J Hum Genet.

[21] Slaugenhaupt SA, Blumenfeld A, Gill SP, Leyne M, Mull J, Cuajungco MP, Liebert CB, Chadwick B, Idelson M, Reznik L, Robbins C, Makalowska I, Brownstein M, Krappmann D, Scheidereit C, Maayan C, Axelrod FB, Gusella JF (2001). Tissue-specific expression of a splicing mutation in the IKBKAP gene causes familial dysautonomia. Am J Hum Genet.

[22] Woloszynska M, Le Gall S, Van Lijsebettens M (2016). Plant Elongator-mediated transcriptional control in a chromatin and epigenetic context. Biochim Biophys Acta.

[23] Nelissen H, Fleury D, Bruno L, Robles P, De Veylder L, Traas J, Micol JL, Van Montagu M, Inze D, Van Lijsebettens M (2005). The elongata mutants identify a functional Elongator complex in plants with a role in cell proliferation during organ growth. Proc Natl Acad Sci U S A.

[24] Chen Z, Zhang H, Jablonowski D, Zhou X, Ren X, Hong X, Schaffrath R, Zhu JK, Gong Z (2006). Mutations in ABO1/ELO2, a subunit of holo-Elongator, increase abscisic acid sensitivity and drought tolerance in Arabidopsis Thaliana. Mol Cell Biol.

[25] Zhou X, Hua D, Chen Z, Zhou Z, Gong Z (2009). Elongator mediates ABA responses, oxidative stress resistance and anthocyanin biosynthesis in Arabidopsis. Plant J.

[26] Xu D, Huang W, Li Y, Wang H, Huang H, Cui X (2012). Elongator complex is critical for cell cycle progression and leaf patterning in *Arabidopsis*. Plant J.

[27] Jia Y, Tian H, Li H, Yu Q, Wang L, Friml J, Ding Z (2015). The *Arabidopsis thaliana* Elongator Complex subunit 2 epigenetically affects root development. J Exp Bot.

[28] Wang C, Ding Y, Yao J, Zhang Y, Sun Y, Colee J, Mou Z (2015). Arabidopsis Elongator subunit 2 positively contributes to resistance to the necrotrophic fungal pathogens *Botrytis cinerea* and *Alternaria brassicicola*. Plant J.

[29] An C, Wang C, Mou Z. The Arabidopsis Elongator complex is required for nonhost resistance against the bacterial pathohen *Xanthomonas citri* subsp. *citri* and *Pseudomonas syringae* pv. *phaseolicola* NPS3121. New Phytol. 2017;214(3):1245–259. 10.1111/nph.14442 28134437

[30] Defraia CT, Wang Y, Yao J, Mou Z (2013). Elongator subunit 3 positively regulates plant immunity through its histone acetyltransferase and radical S-adenosylmethionine domains. BMC Plant Biol.

[31] Wang Y, An C, Zhang X, Yao J, Zhang Y, Sun Y, Yu F, Amador DM, Mou Z. The *Arabidopsis* Elongator complex subunit2 epigenetically regulates plant immune respones. Plant Cell. 2013;25(762–776) 10.1105/tpc.113.109116 PMC3608791 23435660

[32] Zhu M, Li Y, Chen G, Ren L, Xie Q, Zhao Z, Hu Z (2015). Silencing *SlELP2L*, a tomato Elongator complex protein 2-like gene, inhibits leaf growth, accelerates leaf, sepal senescence, and produces dark-green fruit. Sci Rep.

[33] Ding Y, Mou Z (2015). Elongator and its epigenetic role in plant development and responses to abiotic and biotic stresses. Front Plant Sci.

[34] Oosumi T, Gruszewski HA, Blischak LA, Baxter AJ, Wadl PA, Shuman JL, Veilleux RE, Shulaev V (2006). High-efficiency transformation of the diploid strawberry (*Fragaria vesca*) for functional genomics. Planta.

[35] Silva KJP, Brunnings A, Peres NA, Mou Z, Folta KM (2015). The *Arabidopsis NPR1* gene confers broad-spectrum disease resistance in strawberry. Transgenic Res.

[36] Clough SJ, Bent AF (1998). Floral dip: a simplified method for agrobacterium-mediated transformation of *Arabidopsis thaliana*. Plant J.

[37] Verberne MC, Brouwer N, Delbianco F, Linthorst HJ, Bol JF, Verpoorte R (2002). Method for the extraction of the volatile compound salicylic acid from tobacco leaf material. Phytochem Anal.

[38] Shaner G, Finney RE (1977). The effect of nitrogen fertilization on the expression os slow mildewing resistance in Knox wheat. Phytopathology.

[39] Gollner K, Schweizer P, Bai Y, Panstruga R (2008). Natural genetic resources of *Arabidopsis thaliana* reveal a high prevalence and unexpected phenotypic plasticity of RPW8-mediated powdery mildew resistance. New Phytol.

[40] Maas JL, Gouin-Behe C, Hartung JS, Hokanson SC (2000). Sources of resistance for two differentially pathogenic strains of *Xanthomonas fragariae* in Fragaria genotypes. HortSci.

[41] Roberts PD, Jones JB, Chandler CK, Stall RE, Berger RD (1996). Survival of *Xanthomonas fragariae* on strawberry in summer nurseries in Florida detected by specific primers and nested polymerase chain reaction. Plant Dis.

[42] Chang S, Puryear J, Cairney J (1993). A simple and efficient method for isolating RNA from pine trees. Plant Mol Biol Re.

[43] Brunnings AM, Moyer C, Peres NA, Folta KM (2010). Implementation of simple sequence repeat markers to genotype Florida strawberry varieties. Euphytica.

[44] Livak KJ, Schmittgen TD (2001). Analysis of relative gene expression data using real-time quantitative PCR and the 2(−Delta Delta C(T)) method. Methods.

[45] Sehringer B, Zahradnik HP, Deppert WR, Simon M, Noethling C, Schaefer WR (2005). Evaluation of different strategies for real-time RT-PCR expression analysis of corticotropin-releasing hormone and related proteins in human gestational tissues. Anal Bioanal Chem.

[46] Clancy MA, Rosli HG, Chamala S, Barbazuk WB, Civello PM, Folta K (2013). Validation of reference transcripts in strawberry (*Fragaria* spp). Mol Gen Genomics.

[47] Versées W, De Groeve S, Van Lijsebettens M (2010). Elongator, a conserved multitasking complex?. Mol Microbiol.

[48] Huot B, Yao J, Montgomery BL, He SY (2014). Growth-defense tradeoffs in plants: a balancing act to optimize fitness. Mol Plant.

[49] An C, Ding Y, Zhang X, Wang C, Mou Z (2016). Elongator plays a positive role in exogenous NAD-induced defense responses in Arabidopsis. Mol Plant-Microbe Interact.

[50] Turner EL, Malo ME, Pisclevich MG, Dash MD, Davies GF, Arnason TG, Harkness TA (2010). The *Saccharomyces cerevisiae* anaphase-promoting complex interacts with multiple histone-modifying enzymes to regulate cell cycle progression. Eukaryot Cell.

[51] Gu J, Sun D, Zheng Q, Wang X, Yang H, Miao J, Jiang J, Wei W (2009). Human Elongator complex is involved in cell cycle and suppresses cell growth in 293T human embryonic kidney cells. Acta Biochim Biophys Sin Shanghai.

[52] Wittschieben BO, Otero G, de Bizemont T, Fellows J, Erdjument-Bromage H, Ohba R, Li Y, Allis CD, Tempst P, Svejstrup JQ (1999). A novel histone acetyltransferase is an integral subunit of elongating RNA polymerase II holoenzyme. Mol Cell.

[53] Cao H, Glazebrook J, Clark JD, Volko S, Dong X (1997). The Arabidopsis *NPR1* gene that controls systemic acquired resistance encodes a novel protein containing ankyrin repeats. Cell.

[54] Lin WC, CF L, JW W, Cheng ML, Lin YM, Yang NS, Black L, Green SK, Wang JF, Cheng CP (2004). Transgenic tomato plants expressing the Arabidopsis *NPR1* gene display enhanced resistance to a spectrum of fungal and bacterial diseases. Transgenic Res.

[55] Chern M, Fitzgerald HA, Canlas PE, Navarre DA, Ronald PC (2005). Overexpression of a rice NPR1 homolog leads to constitutive activation of defense response and hypersensitivity to light. Mol Plant-Microbe Interact.

[56] Makandar R, Essig JS, Schapaugh MA, Trick HN, Shah J (2006). Genetically engineered resistance to Fusarium head blight in wheat by expression of Arabidopsis NPR1. Mol Plant-Microbe Interact.

[57] Malnoy M, Jin Q, Borejsza-Wysocka EE, He SY, Aldwinckle HS (2007). Overexpression of the apple *MpNPR1* gene confers increased disease resistance in *Malus* x *domestica*. Mol Plant-Microbe Interact.

[58] Yuan Y, Zhong S, Li Q, Zhu Z, Lou Y, Wang L, Wang J, Wang M, Li Q, Yang D, He Z (2007). Functional analysis of rice NPR1-like genes reveals that OsNPR1/NH1 is the rice orthologue conferring disease resistance with enhanced herbivore susceptibility. Plant Biotechnol J.

[59] Meur G, Budatha M, Srinivasan T, Rajesh Kumar KR, Dutta Gupta A, Kirti PB (2008). Constitutive expression of Arabidopsis NPR1 confers enhanced resistance to the early instars of *Spodoptera litura* in transgenic tobacco. Physiol Plant.

[60] Le Henanff G, Heitz T, Mestre P, Mutterer J, Walter B, Chong J (2009). Characterization of *Vitis vinifera* NPR1 homologs involved in the regulation of pathogenesis-related gene expression. BMC Plant Biol.

[61] Wally O, Jayaraj J, Punja ZK (2009). Broad-spectrum disease resistance to necrotrophic and biotrophic pathogens in transgenic carrots (*Daucus carota* L.) expressing an Arabidopsis NPR1 gene. Planta.

[62] Zhang X, Francis MI, Dawson WO, Graham JH, Orbovic V, Triplett EW, Mou Z (2010). Overexpression of the Arabidopsis *NPR1* gene in citrus increases resistance to citrus canker. Eur J Plant Pathol.

[63] Le Henanff G, Farine S, Kieffer-Mazet F, Miclot AS, Heitz T, Mestre P, Bertsch C, Chong J (2011). *Vitis vinifera VvNPR1.1* is the functional ortholog of *AtNPR1* and its overexpression in grapevine triggers constitutive activation of *PR* genes and enhanced resistance to powdery mildew. Planta.

[64] Howard CM, Maas JL, Chandler CL, Albregts EE (1992). Anthracnose of strawberry caused by the *Colletotrichum* complex in Florida. Plant Dis.

[65] Jordan VWL, Hunter T (1972). The effects of glass cloche and coloured polyethylene tunnels on microclimate, growth, yield and disease severity of strawberry plants. J Hortic Sci.

[66] Kennedy BW, King TH (1962). Angular leaf spot of strawberry caused by Xanthomonas fragariae sp. Phytopathology.

