# Supplementary material for: The Arabidopsis ELP3/ELO3 and ELP4/ELO1 genes enhance disease resistance in Fragaria vesca L
Source: BMC Plant Biol. 2017 Dec 1;17:230. doi: 10.1186/s12870-017-1173-5 (PMC5709926; doi:10.1186/s12870-017-1173-5)
Supplement: Additional file 1: Figure S1. — Annotated and correct FvELP3 CDS. Figure S2. Amino acid alignments between AtELPs and FvELPs. Table S1. Primers used in this study. (DOCX 260 kb) [file 12870_2017_1173_MOESM1_ESM.docx]

**Additional file**

**Annotated *FvELP3* CDS**

ATGGCGACTGCGGTGGTGGCGGCGGCAGCCGATCCCATCCGTAAGCAACCCCGTCCGGGCCGGGGCGGGTTCGAAGCCCACGGACTCTCCGAAGAAGAGGCCCGCGTCCGCGCCATAGCCGAAATCGTCGGCTCCATGGTCGACCTCTCCCGCCGAGGCGAAACCGTCGACCTAAACGCCCTCAAAACCGCCGCGTGCCGCAAGTACGGCCTCGCACGTGCACCGAAGCTGGTCGAGATGATCGCCGCGCTTCCGGAATCGGACCGCGAGGCGCTGCTTCCGAAGCTGAAAGCCAAGCCGGTCCGAACCGCCTCCGGAATCGCCGTCGTGGCCGTGATGTCGAAGCCTCACCGGTGCCCTCACATCGCCACCACCGGGAATATTTGCGTGTACTGCCCCGGTGGGCCCGATTCGGATTTTGAGTATAGTACTCAGTCTTATACTGGATACGAGCCTACTAGCATGCGCGCAATTCGAGCCAGATACAACCCGTATGTTCAGGCTAGAGGTAGGATAGATCAGCTGAAGCGATTGGGTCACAGTGTAGACAAGGTTGAGTACATTTTAATGGGTGGTACATTCATGTCGTTGCCAGCAGATTACCGTGATTACTTTATTAGAAATCTTCATGACGCATTATCCGGCCATACTTCTGCCAATGTTGAAGAGGCAGTTGCATACTCTGAGCATGGGGCTACTAAATGTATTGGAATGACCATTGAAACGAGGCCAGATTACTGCCTTGGACCTCACTTGAGACAAATGCTTACTTATGGTTGTACACGATTGGAGATTGGAGTTCAAAGTACATATGAGGATGTGGCTCGTGATACTAATAGAGGTCACACAGTAGCTGCAGTGGCTGATTGTTTCTGCTTGGCAAAGGATGCTGGTTTCAAGGTTGTTGCACACATGATGCCTGATCTTCCAAATGTAGGGGTTGAGAGAGACATGGAAAGCTTTCGTGAATTTTTTGAGAGCCCCTTGTTCAGAGCAGATGGACTTAAAATTTATCCAACACTTGTAATCCGTGGAACTGGACTTTATGAGCTCTGGAAAACTGGAAGGTATAGAAATTACCCACCTGAGCAACTTGTGGACATAGTAGCCAGAATCCTAGCCATGGTACCCCCTTGGACACGTGTTTATAGAGTTCAGAGGGACATTCCAATGCCTCTAGTTACTTCTGGAGTAGAGAAAGGAAACCTTCGGGAGCTTGCTTTAGCTCGGATGGAAGACTTGGGCTTAAAGTGTCGTGATGTTCGAACACGAGAAGCTGGAATTCAGGACATACATCACCAAATTAAGCCGGATGAAGTGGAACTTGTTCGTCGTGATTATACAGCAAATGAAGGTTGGGAAACATTTCTTTCATATGAAGATACACGCCAGGATATTCTAGTTGGGTTGTTGCGATTAAGAAAATGCGGCAGAAATACTTCCTGTCCTGAACTCATGGGGAAGTGTTCTATTGTTCGTGAACTTCATGTTTATGGGACTGCTGTTCCAGTTCATGGGCGGGATGCTGGCAAGCTGCAGCACCAGGGCTACGGTACACTTTTGATGGAAGAAGCAGAGCGAATTGCTAGAAGGGAGCACAGATCAACCAAGATCGCTGTTATTTCAGGAGTAGGAACTCGTCACTACTACAGAAAATTAGGATATGAACTTGAAGGGCCATACATGCCACTATTTACCTTAGTCATGCTAAAGTCAACTTTCACACCCTTATTCTCAACCATTCCCGCCAAACCAAGACTCCCAATACCTTTGCTCAGAGCCTTCACACAAAGCCCACCACCTGTTCGACAAAATGCCTTACTCGCCGTTCACCCACCTCCTACTTCCGCCTACGTCCACCTCCCCTTCTGCCGCAAACGCTGCCACTACTGTGACTTCACCATCATCGCTCTCGGCTCTTCTTCTTCTTCCTCAAACCAACCCCAAGGCAACGACCCGCGAATGGTGAACTATGTCCAGTTACTCTGCCGTGAAATTAATGCAATAAAAGCAGAACATAAAACCAACCCGCCTCTCGAAACTGTCTTTTTCGGAGGCGGCACGCCGTCTCTTGTGCCACCAAAGCTTGTGTCTTCCATTTTGGAGACGCTGAGGTTGAAATTCGGGCTGAGCTCGGATGCTGAGATATCTATGGAAATGGACCCCGGGACTTTCGATGGTGAAAAAATGAAGCGAATGATGGAGTTGGGTGTGAATAGAGTGTCATTGGGAGTTCAGGCGTTTCAACAGGAGCTGTTGAAGTCTTGTGGGAGAGCTCATGGTATTAAGGAGGTTTATGAAGCAATTGAGATTGTTGGGTCATGTGGGGTTGAGAATTGGAGTATGGATCTTATCTCTTCTCTTCCTCATCAGACCCCGGAAATGTGGGAAGAGAGCTTGAGGCTAACAGTGGAAGCTCGTCCTCCCCATGTGTCGGTTTACGATTTGCAAGTGGAACAAGGCACCAAATTCGGAAGATTGTATACGCCAGGGGAGTTCCCGTTGCCTTCTGAAACAAGGTCAGCTGAGTTCTATAGAACGGCATCTCGGACGCTTTGTGATGCAGGTTACGAGCACTATGAGATTAGTAGCTATGGCAAGAGTGGGTTTCAGTGCAAGCATAATCTTACTTACTGGAAGAACAAGCCTTTCTATGGTTTTGGCCTCGGCGCTGCTAGCCATGTTGGTCGTGTGAGGTTTTCGAGACCCAAAATGATGAAAGAGTACAGTGGTTATGTGGAGAATCTGGAAAATGGGTTGGTGGAATGTGGTGGGAGGGATCTCATTGATGCTAAGGATGTGGCCATGGATGTTGTTATGCTGTCTCTAAGAACTTCAAGAGGTCTGGATTTGAAGTGTTTTGGAGAAGCATATGGTAGCTCCCTTGTTGGTTCTCTCTGCGAGGTTTATAAACCTTATGTTGAAAGTGGGCACGTAGTTTTCTTAGATGAACAGAGAAGAGCCATGACTGCGGATGAACTGAACGCCTTGACACTAAACGAAGAAGAGATCGAGCAAATAATTGCCTACATTCGTCTAAGTGATCCAGATGGTTTCCTTTTATCGAATGAATTGATATCCCTTGCGTTTGCAGTTGTATCTCCGTAA

**Correct *FvELP3* CDS**

ATGGCGACTGCGGTGGTGGCGGCGGCAGCCGATCCCATCCGTAAGCAACCCCGTCCGGGCCGGGGCGGGTTCGAAGCCCACGGACTCTCCGAAGAAGAGGCCCGCGTCCGCGCCATAGCCGAAATCGTCGGCTCCATGGTCGACCTCTCCCGCCGAGGCGAAACCGTCGACCTAAACGCCCTCAAAACCGCCGCGTGCCGCAAGTACGGCCTCGCACGTGCACCGAAGCTGGTCGAGATGATCGCCGCGCTTCCGGAATCGGACCGCGAGGCGCTGCTTCCGAAGCTGAAAGCCAAGCCGGTCCGAACCGCCTCCGGAATCGCCGTCGTGGCCGTGATGTCGAAGCCTCACCGGTGCCCTCACATCGCCACCACCGGGAATATTTGCGTGTACTGCCCCGGTGGGCCCGATTCGGATTTTGAGTATAGTACTCAGTCTTATACTGGATACGAGCCTACTAGCATGCGCGCAATTCGAGCCAGATACAACCCGTATGTTCAGGCTAGAGGTAGGATAGATCAGCTGAAGCGATTGGGTCACAGTGTAGACAAGGTTGAGTACATTTTAATGGGTGGTACATTCATGTCGTTGCCAGCAGATTACCGTGATTACTTTATTAGAAATCTTCATGACGCATTATCCGGCCATACTTCTGCCAATGTTGAAGAGGCAGTTGCATACTCTGAGCATGGGGCTACTAAATGTATTGGAATGACCATTGAAACGAGGCCAGATTACTGCCTTGGACCTCACTTGAGACAAATGCTTACTTATGGTTGTACACGATTGGAGATTGGAGTTCAAAGTACATATGAGGATGTGGCTCGTGATACTAATAGAGGTCACACAGTAGCTGCAGTGGCTGATTGTTTCTGCTTGGCAAAGGATGCTGGTTTCAAGGTTGTTGCACACATGATGCCTGATCTTCCAAATGTAGGGGTTGAGAGAGACATGGAAAGCTTTCGTGAATTTTTTGAGAGCCCCTTGTTCAGAGCAGATGGACTTAAAATTTATCCAACACTTGTAATCCGTGGAACTGGACTTTATGAGCTCTGGAAAACTGGAAGGTATAGAAATTACCCACCTGAGCAACTTGTGGACATAGTAGCCAGAATCCTAGCCATGGTACCCCCTTGGACACGTGTTTATAGAGTTCAGAGGGACATTCCAATGCCTCTAGTTACTTCTGGAGTAGAGAAAGGAAACCTTCGGGAGCTTGCTTTAGCTCGGATGGAAGACTTGGGCTTAAAGTGTCGTGATGTTCGAACACGAGAAGCTGGAATTCAGGACATACATCACCAAATTAAGCCGGATGAAGTGGAACTTGTTCGTCGTGATTATACAGCAAATGAAGGTTGGGAAACATTTCTTTCATATGAAGATACACGCCAGGATATTCTAGTTGGGTTGTTGCGATTAAGAAAATGCGGCAGAAATACTTCCTGTCCTGAACTCATGGGGAAGTGTTCTATTGTTCGTGAACTTCATGTTTATGGGACTGCTGTTCCAGTTCATGGGCGGGATGCTGGCAAGCTGCAGCACCAGGGCTACGGTACACTTTTGATGGAAGAAGCAGAGCGAATTGCTAGAAGGGAGCACAGATCAACCAAGATCGCTGTTATTTCAGGAGTAGGAACTCGTCACTACTACAGAAAATTAGGATATGAACTTGAAGGGCCATACATGGTTAAATATCTTGACTAG

**Figure S1.** Annotated and correct *FvELP3* CDS.

The *AtELP3* CDS was used to identify the correct *FvELP3* CDS in *F. vesca* genomic sequence. The nucleotides labeled in red in the annotated *FvELP3* CDS are misannotated. The last 18 nucleotides in the correct *FvELP3* (labeled in blue) were found in the *F. vesca* genomic sequence using *AtELP3* CDS as the query sequence.

**Alignments between AtELP1 (length 1319) and FvELP1 (length=1322)**

Score = 1603 bits (4150), Expect = 0.0, Method: Compositional matrix adjust.

Identities = 814/1336 (61%), Positives = 1016/1336 (76%), Gaps = 29/1336 (2%)

AtELP1 1 MKNLKLFSEVPQNIQLHST--EEVVQFAAYDIDQSRLFFASSANFVYALQLSSFQNESAG 58

M NLKL+SE N+QLHS EE++ F+A+DI+QSRLFFASSAN +Y+ LSS Q+E A

FvELP1 1 MNNLKLYSEASLNLQLHSPQGEELILFSAFDIEQSRLFFASSANNIYSTHLSSLQHERAW 60

AtELP1 59 AKSAMPVEVCSIDI-EPGDFITAFDYLAEKESLLIGTSHGLLLVHNVESDVTELVGNIEG 117

+K+++ +V I++ E DFIT+F YL EKE+LL+GTS GLLL+H+V+ + +++VG ++G

FvELP1 61 SKTSIAAQVSRIELDEAEDFITSFVYLMEKEALLVGTSKGLLLLHSVDENGSQVVGGVDG 120

AtELP1 118 GVKCISPNPTGDLLGLITGLGQLLVMTYDWALMYEKALGEVPEGGYVRETNDLSVNCGGI 177

GV+C+S +P GDL+ +ITG GQ+LVMT DW L+YE AL +V E G S C +

FvELP1 121 GVRCVSASPDGDLVAIITGSGQILVMTLDWDLLYETALEDVAEDG--------STVCDPV 172

AtELP1 178 ------SISWRGDGKYFATMGEVYESGC-MSKKIKIWESDSGALQSSSETKEFTQGILEW 230

I+WRGDGKYF T+ E +S + K++K+WE +SG L + SE+K+F +++W

FvELP1 173 LSNIESPIAWRGDGKYFVTLSEALDSSSSLLKRLKVWERNSGELHAVSESKQFMGSVVDW 232

AtELP1 231 MPSGAKIAAVYKRKSDDSSPSIAFFERNGLERSSFRIGEPEDATESCENLKWNSASDLLA 290

MPSGAK+AAVY RK+ + P+I F+ERNGLERS F I E +AT E LKWN +SDLLA

FvELP1 233 MPSGAKVAAVYDRKAQNECPAIVFYERNGLERSMFSINEQVNAT--VEFLKWNCSSDLLA 290

AtELP1 291 GVVSCKTYDAIRVWFFSNNHWYLKQEIRYPREAGVTVMWDPTKPLQLICWTLSGQVSVRH 350

+V C YD +++W+FSNNHWYLK E RYPR GV +W+PT+PLQLICWTL GQ++ +

FvELP1 291 AIVRCDNYDCVKIWYFSNNHWYLKSEFRYPRHDGVRFVWNPTRPLQLICWTLGGQITSYN 350

AtELP1 351 FMWVTAVMEDSTAFVIDNSKILVTPLSLSLMPPPMYLFSLSFSSAVRDIAYYSRNSKNCL 410

F+W +AVM+DSTA VID+SKILVTPLSL LMPPPMYLFSL F S VRD A+YS+NSKNCL

FvELP1 351 FIWNSAVMDDSTALVIDDSKILVTPLSLCLMPPPMYLFSLKFMSVVRDFAFYSKNSKNCL 410

AtELP1 411 AVFLSDGNLSFVEFPAPNTWEDLEGKDFSVEISDCKTALGSFVHLLWLDVHSLLCVSAYG 470

A FLSDG L VE PA +TWEDLEGK+F VE S + GS +HL+WLD H +L VS +G

FvELP1 411 AAFLSDGCLCVVELPATDTWEDLEGKEFPVEASSSDSPFGSVLHLIWLDPHKILAVSHHG 470

AtELP1 471 SSHNKCLSSGGYDTELHGSYLQEVEVVCHEDHVPDQVTCSGFKASITFQTLLESPVLALA 530

SH+ LS E G YLQE+E+ C EDHVP +TCSGF A ++ + LE + +A

FvELP1 471 FSHSNYLSQSSLGEEDLGFYLQEIELSCSEDHVPGLLTCSGFNAKVSSRNSLEETITGIA 530

AtELP1 531 WNPSKRDSAFVEFEGGKVLGYASRSEIMETRSSDDSVCFPSTCPWVRVAQVDASGVHKPL 590

NP+ + SAFV+F+GGKV Y + I S D F STCPW+ V V S KPL

FvELP1 531 PNPASKGSAFVQFDGGKVYEYVPKLGISRGASKHD-WSFSSTCPWMSVVLVGDSVSSKPL 589

AtELP1 591 ICGLDDMGRLSINGKNLCNNCSSFSFYSELANEVVTHLIILTKQDFLFIVDTKDVLNGDV 650

+ GLDD RL ++ K +CNNCSSFSFYS LA++V+THLI+ TKQD LF+V+ DVL ++

FvELP1 590 LFGLDDSCRLHVSRKIICNNCSSFSFYSNLADQVITHLILATKQDLLFVVEISDVLQKEL 649

AtELP1 651 ALGNVFFVIDGRRRDEENMSYVNIWERGAKVIGVLNGDEAAVILQTMRGNLECIYPRKLV 710

+ + F+ G+++ EEN +++N+WERGAKV+GV++GDEAAV+LQ RGNLECIYPRKLV

FvELP1 650 EIKHENFIHAGKKKREENRNFINMWERGAKVVGVVHGDEAAVLLQPSRGNLECIYPRKLV 709

AtELP1 711 LSSITNALAQQRFKDAFNLVRRHRIDFNVIVDLYGWQAFLQSAVAFVEQVNNLNHVTEFV 770

L+SI NAL Q+RF+DA +VRR RIDFNV+VD GWQ FLQSA FV+QVNNLNH+TEFV

FvELP1 710 LASICNALVQRRFRDALLMVRRQRIDFNVLVDYCGWQVFLQSAAEFVKQVNNLNHMTEFV 769

AtELP1 771 CAMKNEDVTETLYKKFSFSKKGDEVFRVKDSCS------NKVSSVLQAIRKALEEHIPES 824

CA+KNED TETLYK+F E V+ S NKVSSVL AIRKALE+ +PE+

FvELP1 770 CAIKNEDTTETLYKEFISLPSPKEAKDVQSHDSKGSDSNNKVSSVLLAIRKALEDQLPET 829

AtELP1 825 PSRELCILTTLARSDPPAIEESLLRIKSVREMELLNSSDDIRKKSCPSAEEALKHLLWLL 884

P+RELCILTTLARS+PPAI+E+L RIK++RE EL SSD+ R+ S PSAEEALKHLLWL

FvELP1 830 PARELCILTTLARSEPPAIDEALERIKAIREAELSGSSDE-RRMSYPSAEEALKHLLWLS 888

AtELP1 885 DSEAVFEAALGLYDLNLAAIVALNSQRDPKEFLPYLQELEKMPESLMHFKIDIKLQRFDS 944

DSE+VFEAALGLYDLNLAA+VALNSQRDPKEFLP+LQELEKMPE+LM + ID++LQRF+

FvELP1 889 DSESVFEAALGLYDLNLAAMVALNSQRDPKEFLPFLQELEKMPETLMRYNIDLRLQRFEK 948

AtELP1 945 ALRNIVSAGVGYFPDCMNLIKKNPQLFPLGLLLITDPEKKLVVLEAWADHLIDEKRFEDA 1004

AL++IVSAG + D MNL+KKNPQLFPLGL LI DP KK+ VL+AW DHL +EK +EDA

FvELP1 949 ALKHIVSAGDTCYADSMNLMKKNPQLFPLGLQLIADPNKKIQVLDAWGDHLSNEKCYEDA 1008

AtELP1 1005 ATTYLCCCKLEKASKAYRECGDWSGVLRVGALMKLGKDEILKLAYELCEEVNALGKPAEA 1064

A TY+CC EKA K+YR CG+WS VL V ++KLGKDEI++LA+ELCEE+ ALGKP EA

FvELP1 1009 AVTYMCCSSFEKALKSYRSCGNWSKVLTVAGILKLGKDEIMQLAHELCEELQALGKPKEA 1068

AtELP1 1065 AKIALEYCSDISGGISLLINAREWEEALRVAFLHTADDRISVVKSSALECASGLVSEFKE 1124

AKI LEYC DI+ G+SLLI+AR+WEEALRVA +H D IS VK++ALECA L+ E++E

FvELP1 1069 AKIELEYCGDINNGMSLLISARDWEEALRVALMHNRQDLISEVKNAALECAVVLIGEYEE 1128

AtELP1 1125 SIEKVGKYLTRYLAVRQRRLLLAAKLKSEERSVVDLDDDTASEASSNLSGMSAYTLGTRR 1184

+EKVGKYL RYL +RQRRLLLAAKL+SEERS+ DLDDDTASEASSN SGMSAYT GTR+

FvELP1 1129 GLEKVGKYLARYLGLRQRRLLLAAKLQSEERSMNDLDDDTASEASSNFSGMSAYTTGTRK 1188

AtELP1 1185 GSAASVSSSNATSRARDLRRQRKSGKIRAGSAGEEMALVDHLKGMRMTDGGKRELKSLLI 1244

SA S+ SS ATSRARD RRQRK GKIRAGS GEE+ALVDHLKGM T +ELKSLL

FvELP1 1189 SSATSMRSS-ATSRARDARRQRKKGKIRAGSPGEELALVDHLKGMPPTTEALQELKSLLH 1247

AtELP1 1245 CLVTLGEMESAQKLQQTAENFQVSQVAAVELAHDTVSSESVDEEVYCFERYAQKTRSTAR 1304

LV LGE+E+A+KLQ+ ENFQ+S +AAV+LA DTVS++ +DE E Y Q RS +

FvELP1 1248 TLVMLGEVETARKLQKAGENFQLSHMAAVKLAEDTVSTDGIDEHTQTLEHYTQSIRSVVQ 1307

AtELP1 1305 DSDAFSWMLKVFISP* 1320

+S+AF W KVF+SP*

FvELP1 1308 NSEAFFWRCKVFLSP* 1323

**Alignments between AtELP2 (length 838) and FvELP2 (length=840)**

Score = 1135 bits (2935), Expect = 0.0, Method: Compositional matrix adjust.

Identities = 560/842 (67%), Positives = 663/842 (79%), Gaps = 19/842 (2%)

AtELP2 4 NTKVEAKRVFIGAGCNRVVNNVSWGASGLVSFGAQNAVAVFCPKTAQILTTLPGHKASVN 63

T VE KRVFIGAGCNR+VNNVSWGA LV+FGAQNAVAVF PKTAQI TTLPGHKASVN

FvELP2 9 QTDVEVKRVFIGAGCNRIVNNVSWGACDLVAFGAQNAVAVFDPKTAQISTTLPGHKASVN 68

AtELP2 64 CTHWLPTSKFAFKAKKLDRQYLLSGDSDGIIILWELSTLNNDWRHVLQLPLSHKKGVTCI 123

CT WLP++KFAFKAK LD+ YLLSGD+ G IILWE + L WR+VLQ+P HKKGVTCI

FvELP2 69 CTQWLPSNKFAFKAKDLDQHYLLSGDAGGAIILWEYTVLEGKWRYVLQIPELHKKGVTCI 128

AtELP2 124 TAYMVSETDAMFASASSDGVVNVWDVSFPSQPSEECKVVCLDSICVDTKAIVTLSLAELP 183

+ +VS+T+A+FAS SSDG V +W+V FP+ +CK++ LDS+ V +K +V LSLAELP

FvELP2 129 SGILVSDTEAVFASTSSDGTVYIWEVVFPTTGGGDCKLLHLDSLFVGSKPMVALSLAELP 188

AtELP2 184 QNPGRFALALGGLDNKIKLYSGERTGKFTSVCELKGHTDWIRSLDFSLPLHTTEEIPNSI 243

N G LA+GGLDNKI LYSGER GKF CELKGH DWIRSLDFSLP+ E N I

FvELP2 189 GNTGHLVLAMGGLDNKIHLYSGERRGKFVRACELKGHADWIRSLDFSLPIFNNGEAHN-I 247

AtELP2 244 MLVSSSQDKVIRIWKLVLVGDVGSWRR----EITLASYIEGPVFVSGTFTYQISVESVLI 299

+LVSSSQDK IRIWK+ L G + S + +I+LASYIEGPV V+GT +YQIS+ES+LI

FvELP2 248 LLVSSSQDKGIRIWKMALRGSLDSSQSSKPGKISLASYIEGPVLVAGTTSYQISLESLLI 307

AtELP2 300 GHEDWVYSVEWQPPVIDFIDGRLVNHQPLSILSASMDKTMMIWRPEKKTGVWVNVVCVGE 359

GHEDWVYSVEWQPP DG + Q SILSASMDKTMMIW+PEK +G+W+NVV VGE

FvELP2 308 GHEDWVYSVEWQPPSPVSSDG-IAYCQHQSILSASMDKTMMIWKPEKTSGIWMNVVTVGE 366

AtELP2 360 LSHCALGFYGGHWSPNSLSILAHGYGGAFHLWRNVSSSKESENWQMQKVPSGHFAAVTDV 419

LSHCALGFYGGHWSPN SILAHGYGG+FHLWRNV + +NWQ QKVPSGHFAA+TD+

FvELP2 367 LSHCALGFYGGHWSPNGDSILAHGYGGSFHLWRNVGTGL--DNWQPQKVPSGHFAAITDI 424

AtELP2 420 TWARTGEYLLSVSQDQTTRVFSAWKNDEGNEAEDEHWHELARPQVHGHDINCVAMVQGKG 479

W R+GEYLLSVS DQTTR+FS W+N E + ++ WHE+ARPQVHGHD+NCV ++QGKG

FvELP2 425 AWGRSGEYLLSVSDDQTTRIFSPWQN-ETSLGDEGSWHEIARPQVHGHDMNCVTIIQGKG 483

AtELP2 480 NHRFVSGAEEKVVRVFEAPLSFLKTLNHTCAGGEGSFPEDLQADVQVLGANMSALGLSQK 539

NHRFVSGA+EKV RVFEAPLSFLKTL H + +F ED+Q VQ+LGANMSALGLSQK

FvELP2 484 NHRFVSGADEKVARVFEAPLSFLKTLGHAIS-QNSTFSEDIQLGVQILGANMSALGLSQK 542

AtELP2 540 PIYLHSSSEPLERNGGGEGLDTFETVPEAAPAELKEPPIEDQLAFHTLWPESHKLYGHGN 599

PIY+H+ +E+N + LDT E +P+A P L EPPIEDQL +HTLWPESHKLYGHGN

FvELP2 543 PIYVHAEQHTIEKN-PNDSLDTLEAIPDAVPVVLTEPPIEDQLGWHTLWPESHKLYGHGN 601

AtELP2 600 ELFSLCSDHKGNLVASSCKAQSASMAEIWLWEVGTWKAVGRLQSHSLTVTHLEFSYDDTL 659

ELF+LCSDH+G LVASSCKAQSA++AEIWLWEVG+WKAVGRLQSHSLTVT +EFS DD

FvELP2 602 ELFALCSDHEGKLVASSCKAQSAAVAEIWLWEVGSWKAVGRLQSHSLTVTQMEFSLDDKF 661

AtELP2 660 LLSVSRDRHFSVFSIQRTDNGEVSHKLMAKVEAHKRIIWACSWNPFGHQFATSSRDKTVK 719

LL+VSRDR FS+FSI +T S+KL+AK EAHKRIIW+CSWNP G++FAT SRDKTVK

FvELP2 662 LLAVSRDRQFSIFSIDKTGTDGTSYKLVAKHEAHKRIIWSCSWNPHGYEFATGSRDKTVK 721

AtELP2 720 IWSVENDARIKQILVLPPFGSSVTAVAWTGLDRNEKSGCVAVGMESGLIELSNVKIIETE 779

IW+V D+ +K ++ LP F SSVT+++W GLD + +G +AVGMESGLIEL ++ + T+

FvELP2 722 IWTVGKDSSVKLLMTLPQFSSSVTSLSWAGLDSKKNNGVLAVGMESGLIELWSLSVNRTD 781

AtELP2 780 EGTTAT--AALALRLEPFMCHVSAVNRLAW--RPTEKCESNQSLRWLTSCGDDNCVRVFN 835

+G A A L R +P MCHVS+V+RLAW R + C S Q L SCG D+CVRVF

FvELP2 782 DGVAANVLATLVARFDPLMCHVSSVSRLAWRKRKNKDCTSIQ----LASCGADHCVRVFE 837

AtELP2 836 FK 837

K

FvELP2 838 VK 839

**Alignments between AtELP3 (length 565) and FvELP3 (length=567)**

Score = 1077 bits (2785), Expect = 0.0, Method: Compositional matrix adjust.

Identities = 511/554 (92%), Positives = 538/554 (97%), Gaps = 0/554 (0%)

AtELP3 11 LKKQPRPGKGGYQGRGLTEEEARVRAISEIVSTMIERSHRNENVDLNAIKTAACRKYGLA 70

++KQPRPG+GG++ GL+EEEARVRAI+EIV +M++ S R E VDLNA+KTAACRKYGLA

FvELP3 13 IRKQPRPGRGGFEAHGLSEEEARVRAIAEIVGSMVDLSRRGETVDLNALKTAACRKYGLA 72

AtELP3 71 RAPKLVEMIAALPDSERETLLPKLRAKPVRTASGIAVVAVMSKPHRCPHIATTGNICVYC 130

RAPKLVEMIAALP+S+RE LLPKL+AKPVRTASGIAVVAVMSKPHRCPHIATTGNICVYC

FvELP3 73 RAPKLVEMIAALPESDREALLPKLKAKPVRTASGIAVVAVMSKPHRCPHIATTGNICVYC 132

AtELP3 131 PGGPDSDFEYSTQSYTGYEPTSMRAIRARYNPYVQARSRIDQLKRLGHSVDKVEFILMGG 190

PGGPDSDFEYSTQSYTGYEPTSMRAIRARYNPYVQAR RIDQLKRLGHSVDKVE+ILMGG

FvELP3 133 PGGPDSDFEYSTQSYTGYEPTSMRAIRARYNPYVQARGRIDQLKRLGHSVDKVEYILMGG 192

AtELP3 191 TFMSLPAEYRDFFIRNLHDALSGHTSANVEEAVAYSEHSATKCIGMTIETRPDYCLGPHL 250

TFMSLPA+YRD+FIRNLHDALSGHTSANVEEAVAYSEH ATKCIGMTIETRPDYCLGPHL

FvELP3 193 TFMSLPADYRDYFIRNLHDALSGHTSANVEEAVAYSEHGATKCIGMTIETRPDYCLGPHL 252

AtELP3 251 RQMLIYGCTRLEIGVQSTYEDVARDTNRGHTVAAVADCFCLAKDAGFKVVAHMMPDLPNV 310

RQML YGCTRLEIGVQSTYEDVARDTNRGHTVAAVADCFCLAKDAGFKVVAHMMPDLPNV

FvELP3 253 RQMLTYGCTRLEIGVQSTYEDVARDTNRGHTVAAVADCFCLAKDAGFKVVAHMMPDLPNV 312

AtELP3 311 GVERDMESFKEFFESPSFRADGLKIYPTLVIRGTGLYELWKTGRYRNYPPEQLVDIVARI 370

GVERDMESF+EFFESP FRADGLKIYPTLVIRGTGLYELWKTGRYRNYPPEQLVDIVARI

FvELP3 313 GVERDMESFREFFESPLFRADGLKIYPTLVIRGTGLYELWKTGRYRNYPPEQLVDIVARI 372

AtELP3 371 LSMVPPWTRVYRVQRDIPMPLVTSGVEKGNLRELALARMDDLGLKCRDVRTREAGIQDIH 430

L+MVPPWTRVYRVQRDIPMPLVTSGVEKGNLRELALARM+DLGLKCRDVRTREAGIQDIH

FvELP3 373 LAMVPPWTRVYRVQRDIPMPLVTSGVEKGNLRELALARMEDLGLKCRDVRTREAGIQDIH 432

AtELP3 431 HKIKPEQVELVRRDYTANEGWETFLSYEDTRQDILVGLLRLRKCGKNVTCPELMGKCSVV 490

H+IKP++VELVRRDYTANEGWETFLSYEDTRQDILVGLLRLRKCG+N +CPELMGKCS+V

FvELP3 433 HQIKPDEVELVRRDYTANEGWETFLSYEDTRQDILVGLLRLRKCGRNTSCPELMGKCSIV 492

AtELP3 491 RELHVYGTAVPVHGRDADKLQHQGYGTLLMEEAERIARREHRSNKIGVISGVGTRHYYRK 550

RELHVYGTAVPVHGRDA KLQHQGYGTLLMEEAERIARREHRS KI VISGVGTRHYYRK

FvELP3 493 RELHVYGTAVPVHGRDAGKLQHQGYGTLLMEEAERIARREHRSTKIAVISGVGTRHYYRK 552

AtELP3 551 LGYELEGPYMVKHL 564

LGYELEGPYMVK+L

FvELP3 553 LGYELEGPYMVKYL 566

**Alignments between AtELP4 (length 355) and FvELP4-1 (length=362, encoded by gene09242)**

Score = 455 bits (1171), Expect = 7e-166, Method: Compositional matrix adjust.

Identities = 253/364 (70%), Positives = 291/364 (80%), Gaps = 9/364 (2%)

AtELP4 1 MAAPNVRSSSSFSRNISVVSSPQIPGLKSGPNGTAFISSGIRDLDRILGGGYPLGSLVMV 60

MAA R+SS FSRN S SSPQIPGLK GPNGT F+SSGI DLD+ILGGG+ LGSLVMV

FvELP4 1 MAASKTRTSS-FSRNFSGASSPQIPGLKHGPNGTMFVSSGIPDLDKILGGGFALGSLVMV 59

AtELP4 61 MEDPEAPHHMDLLRTYMSQGLVNNQPLLYASPSKDPKGFLGTLPHPASSKEDKPTAPDPD 120

MED EAPHHM LLR +MSQGLV+NQPLLYASP+KDP+ FLGTLP PA K+ K +PD

FvELP4 60 MEDAEAPHHMLLLRNFMSQGLVHNQPLLYASPAKDPRQFLGTLPSPAVPKDGKSGHRNPD 119

AtELP4 121 QGESLRIAWQYRKYL-ENQK-----NAIDDYSNDFDMRKPLERQFLSGRPIDCVSLLDSS 174

Q + LRIAWQY+KY ENQ+ N ++ N+FD+RKPLERQFL+G+ IDC S+LDS

FvELP4 120 QDKGLRIAWQYKKYFGENQQGFDSQNGKHEFCNNFDLRKPLERQFLTGKRIDCASILDSP 179

AtELP4 175 DLSIAQDHCATFLSKFPRNSSNIASIGRIAIQSFCSPLCEYSEKESDMLSFIRLLKSMLM 234

+L D CATFLS+FPRN SNI+ +GRIAIQSFC+P C YS E DMLS +R LKSML

FvELP4 180 NLVTLHDRCATFLSQFPRNDSNISCVGRIAIQSFCAPQCGYSNLEWDMLSLLRSLKSMLR 239

AtELP4 235 VSNAVAIVTFPPSLLSPSSSKRLQHMADTLLSIKAIPDGDKELEKLLTGYKDINGFLNIH 294

SNAVA+VTFPP+LLS SSS R QHMADTLLS+KA+PD DKEL LLTGY+D+ G LN+

FvELP4 240 SSNAVAVVTFPPTLLSSSSSTRWQHMADTLLSVKALPDEDKELATLLTGYQDMVGLLNVQ 299

AtELP4 295 KVARINTQVPVILEAKTFSMSLKKRRFLALECLNQAPVDGSSGTSYGTSGSC--SSKSGALDF* 333

KVA+INTQVP+IL+A TFS+ L+KRRFL LECLNQAPVDGSSG SYGTS SC SSK G LDF*

FvELP4 300 KVAQINTQVPIILDATTFSIKLQKRRFLVLECLNQAPVDGSSGGSYGTSSSCSGSSKTGSLDF* 338

**Alignments between AtELP4 (length 355) and FvELP4-2 (length=373, encoded by gene20701)**

Score = 456 bits (1174), Expect = 4e-166, Method: Compositional matrix adjust.

Identities = 233/339 (69%), Positives = 272/339 (80%), Gaps = 7/339 (2%)

AtELP4 1 MAAPNVRSSSSFSRNISVVSSPQIPGLKSGPNGTAFISSGIRDLDRILGGGYPLGSLVMV 60

MAA R+SS FSRN S SSPQIPGLK GPNGT F+SSGI DLD+ILGGG+ LGSLVMV

FvELP4 1 MAATKTRTSS-FSRNFSGASSPQIPGLKHGPNGTMFVSSGIPDLDKILGGGFALGSLVMV 59

AtELP4 61 MEDPEAPHHMDLLRTYMSQGLVNNQPLLYASPSKDPKGFLGTLPHPASSKEDKPTAPDPD 120

MED EAPHHM LLR +MSQGLV+NQ LLYASP+KDP+ FLGTLP PA K++K + DPD

FvELP4 60 MEDAEAPHHMLLLRNFMSQGLVHNQHLLYASPAKDPRQFLGTLPSPAVPKDEKSSHRDPD 119

AtELP4 121 QGESLRIAWQYRKYL-ENQK-----NAIDDYSNDFDMRKPLERQFLSGRPIDCVSLLDSS 174

Q + LRIAWQY+KY ENQ+ N ++ N+FD+RKPLERQFL+G+ I+C S+LDS

FvELP4 120 QEKGLRIAWQYKKYFGENQQGFDSQNGKHEFCNNFDLRKPLERQFLTGKLIECASILDSP 179

AtELP4 175 DLSIAQDHCATFLSKFPRNSSNIASIGRIAIQSFCSPLCEYSEKESDMLSFIRLLKSMLM 234

+L D CATFLS+FPRN SNI+ +GRIAIQSFC+P C YS E DMLS +R LKSML

FvELP4 180 NLVTFHDRCATFLSQFPRNDSNISCVGRIAIQSFCAPQCGYSNLEWDMLSLLRSLKSMLR 239

AtELP4 235 VSNAVAIVTFPPSLLSPSSSKRLQHMADTLLSIKAIPDGDKELEKLLTGYKDINGFLNIH 294

SNAVA+VTF PSLLS SSS R QH+ADTLLS+KA+PD DKEL LLTGY+D+ G LN+

FvELP4 240 SSNAVAVVTFLPSLLSSSSSTRWQHIADTLLSVKALPDEDKELATLLTGYQDMVGLLNVQ 299

AtELP4 295 KVARINTQVPVILEAKTFSMSLKKRRFLALECLNQAPVD 333

KVA+INTQVPVIL+A TFS+ L+KRRFL LECLNQAPVD

FvELP4 300 KVAQINTQVPVILDATTFSIKLQKRRFLVLECLNQAPVD 338

**Alignments between AtELP5 (length 374) and FvELP5 (length=389)**

Score = 345 bits (885), Expect = 1e-121, Method: Compositional matrix adjust.

Identities = 193/384 (50%), Positives = 246/384 (64%), Gaps = 32/384 (8%)

AtELP5 2 AESIFRKLRDGGEEGELAPALTIEETVASPFGLDVSGYLLTNLSSSILAGKSSSQ----- 56

AE I R +RDG EGE APALTI++T+ASP G V + L+ S++I A KS S+

FvELP5 3 AEWICRAVRDGALEGEHAPALTIKDTMASPLGFHVFTHFLSQQSTNISAAKSQSRFVPLL 62

AtELP5 57 ------------------GLVLITFSRSPSFYLQLLKQKGIVVSSSSKWIRILDCYTDPL 98

GLVL+ SRSPSFYL LL KG+ +S S IRILDCY+DPL

FvELP5 63 QNYAVLRLFHFYSIFICRGLVLVALSRSPSFYLDLLSNKGLDTASLSNKIRILDCYSDPL 122

AtELP5 99 GWIDQSSTSFSEGSSLIKLHKCVSDLKKLFSSIIEAGRELVGTGKTRFCVAIDSVNELLR 158

GW + G S V D+ LFSS+I G LVG GK RFCVAIDSVNE+LR

FvELP5 123 GWRAHHLSDDIAGIS-----GNVKDVDALFSSVISLGTGLVGQGKDRFCVAIDSVNEMLR 177

AtELP5 159 HSAMPLVSGLLTDLRSHAQISSVFWSLNTDLHQEKVTNALEYISTMKANLEPLCPSSDGQ 218

++++ VSGL++ LR QISS+FW + DL +E+VT+ALEY+S+M A++EPL ++GQ

FvELP5 178 YASLSSVSGLISSLRCCGQISSIFWLCHADLCEERVTSALEYMSSMVASIEPLIQFANGQ 237

AtELP5 219 RNALENLFSVHQDFGKGRFHVRFKLRKGRVRVMSEEYHVDQSGINFSPISSVDTVIAATK 278

R+ ENL Q F KG+ H+ K R GRVRVM EE H+ QSGINF+ ISS +I +

FvELP5 238 RSNSENLSLRDQSFTKGKLHLHCKRRNGRVRVMFEEIHIGQSGINFTSISSEGELI--NQ 295

AtELP5 279 SLLPKVQFNLQLSEKERVEKEKVVLPFEHQDDGKSNEIYDGRRSLVDGKIETTPLSSMEL 338

L+PKVQF+LQLSEKE ++ VVLPFEHQ +GK +IYDGR+SL D K E P+ +

FvELP5 296 GLVPKVQFSLQLSEKELKDRANVVLPFEHQGNGKPVQIYDGRKSLTDSKYEVLPVQNG-- 353

AtELP5 339 QTDVVSSGKGGEIIYFRDSDDEHP 362

+D+ GEIIYFRDS+DE P

FvELP5 354 NSDIDKESSKGEIIYFRDSEDEMP 377

**Alignments between AtELP6 (length 262) and FvELP6 (length=253)**

Score = 281 bits (719), Expect = 2e-100, Method: Compositional matrix adjust.

Identities = 145/260 (56%), Positives = 188/260 (72%), Gaps = 12/260 (5%)

AtELP6 6 NLLDLALGFDEQLAIPSPLNGKVILIEDCVETSGSFVLHQLMKRVLSS--NSSDALIFLA 63

+LLD ALG L+G+V+L+EDCVETS +FVLH L+KR LS +SS+ ++F+A

FvELP6 5 SLLDEALGLQH-------LSGRVVLLEDCVETSAAFVLHHLLKRALSQPLHSSNVVVFVA 57

AtELP6 64 FARPFSHYDRILRKLGCNLATHKSNNRLVFFDMLMVKCSDGDQMEDNVSAVAKLFREIQE 123

FA+PFSHYDRILRKLGCNLA + N++ FFDML C GD + + L+ I +

FvELP6 58 FAQPFSHYDRILRKLGCNLAVQRDNSKFFFFDMLRDFCPGGDDGKGGDGGLVSLYGRILK 117

AtELP6 124 TVRKLQSVTSGNITVMVDDMSLLEIATTGSNSDHVLDFLHYCHTLSSESNCSLVILNHED 183

T+ L +TVM+DD+SL+E+A GS ++ VLDFLHYCHTL SE CSLV+LNHED

FvELP6 118 TISALPEENKNRVTVMIDDVSLMEVAAKGS-TNLVLDFLHYCHTLISEFGCSLVMLNHED 176

AtELP6 184 IYASMERPAFLLQMVCLADVVIKAEPLASGLANDVHGQLTVLNKGISNSGRGSSRNKLQN 243

IY+ M RP +LQM LAD++IKAEPLA+GLA+DVHGQLTV N+ I++ R SRNKL N

FvELP6 177 IYSCMGRPTLILQMEYLADILIKAEPLATGLASDVHGQLTVANRSINDGER--SRNKLCN 234

AtELP6 244 FQFRIKENGIDYFYPGCRS* 263

F F++KEN ++YFYPG R+*

FvELP6 235 FHFKVKENSVEYFYPGSRT* 254

**Figure S2.** Amino acid alignments between AtELPs and FvELPs.

The amino acid sequences of AtELPs and FvELPs were entered into the online Align Sequences Protein BLAST tool (https://blast.ncbi.nlm.nih.gov/Blast.cgi?PAGE=Proteins&PROGRAM=blastp&BLAST_PROGRAMS=blastp&PAGE_TYPE=BlastSearch&BLAST_SPEC=blast2seq&DATABASE=n/a&QUERY=&SUBJECTS=) and the alignment results are presented. Note that there are two genes, gene09242 and gene20701, encoding FvELP4 in *F. vesca* and that FvELP4-1 is more similar to AtELP4 than FvELP4-2. The last 24 amino acids of FvELP4-1 (22 in AtELP4) were manually added.

**Table S1.** Primers used in this study.

| **Primers** | **Sequences (5’ to 3’)** |
| --- | --- |
| *AtELP3F* | GTGGATTAACTGAAGAAGAAGCTCG |
| *AtELP3R* | AATCCTCTCTGCTTCTTCCATCA |
| *AtELP4F* | CAAACGTTCGTAGTAGTAGCAGCTT |
| *AtELP4R* | CAAAAATCTAGTGCTCCGGATTTGG |
| *SalI-FvELP4F* | ACGCGTCGACATGGCTGCATCCAAGACTCG |
| *SacI-FvELP4R* | CGAGCTCTAAAAATCAAGAGATCCAGTCTTAG |
| *elF1αF* | GCCCATGGTTGTTGAAACTTT |
| *elF1αR* | GGCGCATGTCCCTCACA |
| *qEF1aF* | GCCCATGGTTGTTGAAACTTT |
| *qEF1aR* | GGC GCATGTCCCTCACA |
| *qPR1F(PR1.2)* | CCAAGACACACCGAAAGACTACCT |
| *qPR1R(PR1.2)* | GACGAGTTGGACATCATCTACGTGAG |
| *qPR5F(PR5.3)* | CGTAGTTAGGTCCACCGAAGCATGTA |
| *qPR5R(PR5.3)* | ACCTCCTAATGACACTCCCGAAACA |
| *qAtELP3F* | CCAGGTAAAGGCGGCTATCA |
| *qAtELP3R* | GGCGCGAACTCGAGCTT |
| *qAtELP4F* | GCTGCACCAAACGTTCGTAGT |
| *qAtELP4R* | GAGGCGATGACACAACTGATATG |
